# Genetic Structure and Evolutionary History of Three Alpine Sclerophyllous Oaks in East Himalaya-Hengduan Mountains and Adjacent Regions

**DOI:** 10.3389/fpls.2016.01688

**Published:** 2016-11-11

**Authors:** Li Feng, Qi-Jian Zheng, Zeng-Qiang Qian, Jia Yang, Yan-Ping Zhang, Zhong-Hu Li, Gui-Fang Zhao

**Affiliations:** ^1^Key Laboratory of Resource Biology and Biotechnology in Western China (Ministry of Education), College of Life Sciences, Northwest UniversityXi'an, China; ^2^College of Life Sciences, Shaanxi Normal UniversityXi'an, China

**Keywords:** East Himalaya-Hengduan Mountains, ecological niche models, environmental heterogeneity, evolutionary history, gene flow, *Quercus*

## Abstract

The East Himalaya-Hengduan Mountains (EH-HM) region has a high biodiversity and harbors numerous endemic alpine plants. This is probably the result of combined orographic and climate oscillations occurring since late Tertiary. Here, we determined the genetic structure and evolutionary history of alpine oak species (including *Quercus spinosa, Quercus aquifolioides*, and *Quercus rehderiana*) using both cytoplasmic-nuclear markers and ecological niche models (ENMs), and elucidated the impacts of climate oscillations and environmental heterogeneity on their population demography. Our results indicate there were mixed genetic structure and asymmetric contemporary gene flow within them. The ENMs revealed a similar demographic history for the three species expanded their ranges from the last interglacial (LIG) to the last glacial maximum (LGM), which was consistent with effective population sizes changes. Effects of genetic drift and fragmentation of habitats were responsible for the high differentiation and the lack of phylogeographic structure. Our results support that geological and climatic factors since Miocene triggered the differentiation, evolutionary origin and range shifts of the three oak species in the studied area and also emphasize that a multidisciplinary approach combining molecular markers, ENMs and population genetics can yield deep insights into diversification and evolutionary dynamics of species.

## Introduction

Historical processes such as geographic and climate changes have profoundly shaped the population genetic structure and demographic history of extant species (Hewitt, 2000). Global cyclical cooling-warming events during the Quaternary Period have resulted in periodic expansions and contractions of the ranges of species. For instance, in western Eurasia, species retreated into southern refuges during glacial episodes and recolonized during warmer interglacials; this periodical process created a frequently observed south-north gradient in genetic diversity (southern richness, northern purity) during postglacial re-expansion (Hewitt, 1996). Other researches have suggested that environmental heterogeneity may also contribute to the genetic differentiation among species or populations (e.g., Ortego et al., 2012, 2015; Guichoux et al., 2013). In China, phylogeographic studies have focus mainly on the roles of historical orogenesis, climatic oscillations and environmental heterogeneity in evolutionary history of biotas in Qinghai-Tibet Plateau (QTP) and adjacent regions (Qiu et al., 2011; Liu et al., 2012; Wen et al., 2014). However, recent study has suggested that the demographic history of flora occurred in the QTP must be revised because the QTP has been 4–5 km high since the mid-Eocene while numerous studies linked young inferred divergence times to recent QTP uplift phases (also see review by Renner (2016) and references therein).

The QTP and adjacent regions have long been considered as biodiversity hotspots with numerous endemic species (Myers et al., 2000). The uplift of QTP as a consequence of the collision of the Indian subcontinent with the Eurasian plate 40–50 million years ago (Yin and Harrison, 2000) and subsequent Quaternary climatic oscillations altered the distribution patterns of organisms as well as affected biodiversity there (Qiu et al., 2011). It is hypothesized that uplifts of the QTP, which started during middle Eocene and continued into middle Pliocene, created conditions for the origin and divergence of multitudinous species in the QTP and adjacent regions (Qiu et al., 2011). During the Quaternary glacial periods, these locations likely became refuges for plants belonging to the North Temperate Zone of East Asia (Wu, 1987; Qiu et al., 2011; Liu et al., 2012). During interglacials, species previously retreated into a refuge might recolonize high-altitude regions and also areas outside the QTP (Qiu et al., 2011, and references therein). Nonetheless, some species in these areas responded to Quaternary climate changes in other ways. For instance, *Taxus wallichiana* and *Picea likiangensis* supposed to originate in the HHM regions experienced ranges expansion during the Last Glacial Maximum (LGM) rather than during the Last Interglacial (LIG) (Li et al., 2013; Liu et al., 2013). It has yet unknown if this unexpected pattern of range shift is valid also for other cold-tolerant trees in this area. In addition, previous studies suggested that the accumulated genetic differences between species due to genetic admixture and introgression might be eroded by a shift of distribution ranges introduced by geological and climatic changes (Du et al., 2011; Ma et al., 2014), however, it remains poorly understood.

The genus *Quercus* comprises 531 species of trees and shrubs, and includes keystone taxa in the temperate and (sub-)tropical areas of the Northern Hemisphere (Nixon, 1993). Hybridization between related oak species is common and frequently genetic admixture has been reported (Aldrich and Cavender-Bares, 2011, and references therein). Weak reproductive isolation, frequent interspecies gene exchange and high phenotypic variation result in diffuse species boundaries within the genus causing much debate about species concepts (Burger, 1975; Rushton, 1993). Up to date, numerous studies in Europe and America have focused on the impact of climate change on their evolutionary history (e.g., Ortego et al., 2012, 2015; Guichoux et al., 2013; Cavender-Bares et al., 2015). Within our scope, few reports concerned oak species in China (Zeng et al., 2010, 2011, 2015), and even less alpine sclerophyllous oaks.

In the present study, we focused on species of the *Quercus* group *ilex* (synonyms *Quercus* subgenus *Heterobalanus*), i.e., *Quercus spinosa, Quercus aquifolioides*, and *Quercus rehderiana* (see details in Note S1). Previous studies based on molecular and morphological evidence of pollen suggested this group corresponded to the subgenus *Heterobalanus* (Manos et al., 2001; Denk and Grimm, 2010; Denk and Tekleva, 2014). However, no information is available on the evolutionary history and demography of them. In this study we use an integrative approach to determine the evolutionary history of the three oak species to infer the most plausible scenario(s) of speciation and their responses to climatic changes. The major objectives of this study were to investigate (i) whether the uplift of the QTP as well as Quaternary climate shifts gave rise to the current differences of the three oak species; (ii) if so, whether they have responded similarly to climatic changes and the uplift of the QTP; and (iii) whether and how the gene flow effected their population genetic structure. Obviously, knowledge of the population structure and evolutionary history of the evergreen oak species in temperate China is needed to understand the complicated evolutionary history of species in the East Himalaya-Hengduan Mountains (EH-HM) and adjacent regions.

## Materials and methods

### Sampling and genotyping

Samples were collected strictly according to morphological description from 608 individual trees in 33 natural populations and one individual of *Q. spinosa* in Taiwan (Table S1), covering the majority of their distribution ranges. Generally, 4–20 samples were collected from each population depending on the population size. All sampled individuals from each population were spaced at least 100 m apart. After DNA extraction, three chloroplast DNA (cpDNA), one nuclear ribosomal internal transcribed spacer (ITS) and 12 nuclear simple sequence repeat (nSSR) microsatellite loci were amplified (see details in Note S2, and Table S10).

### DNA sequence analysis

Population genetic parameters, including the number of segregating sites (*S*), the number of haplotypes (*N*_H_), the haplotype diversity (*H*_d_), the nucleotide diversity (ϕ), and neutrality test statistics of Tajima's *D* (Tajima, 1989) and Fu's (*F*s) (Fu and Li, 1993) to assess possible expansions with associated significance values for species were estimated by DnaSP v5.00.04 (Librado and Rozas, 2009). The program PERMUT v1.2.1 (Pons and Petit, 1996) was employed to estimate the average gene diversity within populations (*H*_S_), total gene diversity (*H*_T_) and the differentiation for unordered alleles (*G*_ST_) and for ordered alleles (*N*_ST_) based on 1000 random permutations.

The phylogenetic relationships of cpDNA haplotypes were reconstructed via Bayesian and maximum likelihood (ML) methods with *Castanea mollissima* as the outgroup, and we utilized the program BEAST v1.7.5 (Drummond et al., 2012) to estimate their divergence times. In tree-related analysis, we employed jMODELTEST v1.0 (Posada, 2008) to evaluate the best-fit model of nucleotide substitution for maximum likelyhood (ML) method to infer haplotype relationships(GTR+I+G in the present case). The program MrBayes v3.1 (Ronquist and Huelsenbeck, 2003) was used for the Bayesian analysis with a burn-in of 3 × 10^6^ Markov chain Monte Carlo (MCMC) repetitions for cpDNA sequences. We also used maximum parsimony (MP) and ML in PAUP^*^ v4.0 beta 10 (Swofford, 2002) to determine the relationships of cpDNA haplotypes and visualized results using FIGTREE v1.3.1 (http://tree.bio.ed.ac.uk/software/figtree/).

The program BEAST v1.7.5 was used to estimate the divergence times of the three related species based on an uncorrelated lognormal relaxed molecular clock model when considering the difference of substitution rates among three cpDNA fragments and the different evolutionary rate along tree branch during their evolutionary history. Three independent runs of 5 × 10^8^ MCMC steps were carried out, with sampling at every 5000 generations, following a burn-in of the initial 10% cycles. MCMC samples were inspected in TRACER v1.5 to confirm sampling adequacy and convergence of the chains to a stationary distribution. The results were visualized using FIGTREE v.1.3.1. Generally, previous studies utilized the cpDNA substitutions rates [1.1 × 10^−9^ to 2.9 × 10^−9^ substitutions per site per year (s/s/y)] (Wolfe et al., 1987) when estimating divergence time, while, Petit and Hampe (2006) suggested that woody plants shared a slow evolutionary clock due to a long generation time. Hence, we estimated the genetic divergence times between the clades by using cpDNA substitution rates of 7.905 × 10^−10^ s/s/y in *Q. variabilis* (Chen et al., 2012).

We also used the locus-by-locus analysis of molecular variance (AMOVA) approach as implemented in ARLEQUIN v3.5 (Excoffier and Lischer, 2010) to examine variance within and between population groups with significance differences on 1000 permutations. The program NETWORK v4.2.0.1 (Bandelt et al., 1999) was used to infer the relationships of haplotypes of cpDNA and rDNA with median-joining networks.

### Microsatellite data analysis

We used MICROCHECKER v2.2.3 (Van Oosterhout et al., 2004) to test the presence of null alleles for all loci. For each microsatellite locus, genetic diversity indices (total number of alleles, *A*_O_, observed heterozygosity, *H*_O_, expected heterozygosity over all populations, *H*_E_, gene diversity within populations, *H*_S_, and total genetic diversity, *H*_T_) were estimated by POPGENE v1.31 (Yeh et al., 1997). Linkage disequilibrium (LD) and departure from Hardy-Weinberg equilibrium (HWE) were also evaluated using FSTAT v2.9.3 (Goudet, 2001). Significance levels were corrected by the sequential Bonferroni method (Rice, 1989).

Genetic differentiation among populations was evaluated using θ (*F*_ST_) (Weir and Cockerham, 1984) and the standardized genetic differentiation *G*'_ST_(Hedrick, 2005) across loci with the program SMOGD (Crawford, 2010). Compared to traditional measures, *G*'_ST_ is a more suitable measure for highly polymorphic markers such as microsatellites. In addition, we performed AMOVA analysis in ARLEQUIN v3.5 for these three related species, respectively. The significance of fixation indices was tested using 10^4^ permutations.

The program STRUCTURE v2.3.3 (Pritchard et al., 2000) was used to determine whether these three named species are genetically distinct based on the Bayesian clustering analysis using the microsatellite data. The analyses were run using the admixture model with independent allele frequencies. A total of 10 independent simulations were run for *K* from 1 to 30 with 1.0 × 10^5^ burn-in steps followed by 2.0 × 10^5^ MCMC steps. Two alternative methods were utilized to estimate the most likely number (*K*) of genetic clusters with the program STRUCTURE HARVSTER (Earl and vonHoldt, 2012), i.e., by tracing the change in the average of log-likelihood L(*K*) (Pritchard et al., 2000) and by calculating delta *K* (Δ*K*) (Evanno et al., 2005). As with the whole dataset, two clusters were identified (**Figure 3**) and we repeated the structure analysis independently except for running K from 1 to 15 for populations occurred in the EH-HM region (green cluster in **Figure 3C**).

In order to detect the relationship between genetic and geographic distances among populations within species, we conducted a Mantel test (Mantel, 1967) with 10,000 permutations to determine the possible role of isolation-by-distance (IBD) in the formation of current population structure using IBDW v3.23 (Jensen et al., 2005).

In order to determine whether there has been subsequent population expansion within the species, we used BOTTLENECK v1.2.02 (Piry et al., 1999) to detect genetic bottlenecks in all populations. First of all, we evaluated any excess in heterozygosity in a population at mutation-drift equilibrium through Wilcoxon sign-rank test with 10^4^ replications. Then, we performed the analysis under stepwise mutation model (SMM) and two-phase model of microsatellite evolution with the proportion of single step stepwise mutation set to 95% and the variance set to 5% with 10^4^ simulations as recommended by Piry et al. (1999). The program 2MOD v0.2 (Ciofi et al., 1999) was used to evaluate the relative likelihood of migration-drift equilibrium, i.e., the relative importance of gene flow vs. genetic drift in forming current population structure. We ran the program six times with each model for all three species; 10^5^ iterations were performed, and the first 10% of the iterations were discarded as burn-in. We used Bayesian factors as calculated by TRACER v1.5 to describe the probability of the most likely model.

To explore the historical and contemporary gene flow within the three oak species, we employed MIGRATE-N v3.6 (Beerli, 2006) and BAYESASS v3.0 (Wilson and Rannala, 2003) to estimate the effective number of migrants (*2Nem*, where *Ne* = effective population size, *m* = migration rates per generation) and contemporary migration rates (over the last few generations, *m*_*c*_), respectively. We defined four clusters as follows: one for *Q. aquifolioides*, one for *Q. rehderiana*, and the other two for *Q. spinosa* as suggested by the results of STRUCTURE (see results section). We performed maximum-likelihood analyses in MIGRATE-N v3.6 using 10 short chains (10^4^ trees) and three long chains (10^5^ trees); 10^4^ trees were discarded as burn-in. To increase the efficiency of the MCMC, we applied the following parameters: replicated = YES, long chains, randomtree = YES, heating = ADAPTIVE: 1 (1, 1.2, 1.5, 3.0). In order to ensure the consistency of estimates, we repeated this procedure five times and reported average maximum-likelihood estimates with 95% confidence intervals. When estimated the contemporary gene flow using BAYESASS v3.0, We examined the parameters including migration rates (Δ*M*), allele frequencies (Δ*A*) and inbreeding coefficients (Δ*F*) to ensure the optimal acceptance rates of the three parameters fell within the range of 20–60%. The delta values for the three parameters were 0.26, 0.37, and 0.49, respectively. Following that, we conducted the analyses with 10^7^ iterations after a burn-in of 10^6^ iterations, setting 1000 as the sampling frequency. Ten independent runs were executed to minimize the convergence problem. The result with the lowest deviance was adopted according to the method of Meirmans (Meirmans, 2014). We estimated the 95% credible interval as mc ± 1.96 • standard deviation.

In order to evaluate the plausible scenario of evolutionary history (i.e., divergent scenario) and population dynamics (i.e., changes in effective population sizes) of these oak species, We used the approximate Bayesian computation (ABC) procedure in DIYABC v2.04 (Cornuet et al., 2014). Firstly, we used a multispecies coalescent model to estimate the species tree based on data of three cpDNA fragments under the Star BEAST (^*^BEAST) option implemented in BEAST v1.7.5. The result of species tree suggested the *Q. spinosa* was more ancient than other two species. Furthermore, Ma (2006) suggested that the *Q. rehderiana* was the youngest species within the three oak species. Additionally, Zhou (1992, 1993) suggested that Chinese oak species originated from the Indo-Chinese region, which implies the migration routs of oak species in this study are west to east. In other words, the oak species or populations occurred in the EH-HM region are more ancient than that in other regions. Hence, based on the above results and the STRUCTURE results (**Figure 3C** and Figure S3), nine alternative scenarios of population history for the four lineages were summarized (**Figure 5A**, Tables S6, S7). We assumed uniform priors on all parameters and used a goodness-of-fit test to check the priors of all parameters before implementing the simulations (Table S7). Each simulation was summarized by the following summary statistics: the mean number of alleles and the mean genic diversity for each lineage, the *F*_ST_, the mean classification index, and shared allele distance between pairs of lineages. To select the model that best explains the evolutionary history observed in these species, 1 million simulated data sets for each scenario were run and total 9 million simulations were run for all scenarios, of which the 1% closest to the observed data was used to estimate the relative posterior probabilities of each scenario via a logistic regression approach and posterior parameter distributions according to the most likely scenario (Cornuet et al., 2008, 2014). In addition, DIYABC was used to simulate and examine demographic changes in the recent past. We tested the following six scenarios of demographic changes of the oak species in this study based on the discordant results of neutrality test of cpDNA and ecological niche modeling (see Results Section): continuous shrinkage or expansion; shrinkage-expansion or expansion-shrinkage; and shrinkage-expansion-shrinkage or expansion-shrinkage-expansion (**Figure 4B** and Table S7). DIYABC allows selection of the demographic scenario that best fits the data and parameters of interest. Summary statistics included the mean number of alleles across loci, the mean gene diversity across loci, and the mean allele size range variation across loci.

### Ecological niche modeling

Ecological niche modeling was employed to predict suitable palaeo- and current distribution ranges of the three species via MAXENT v.3.3.3k (Phillips et al., 2006) and DESKTOP GARP v1.1.6 (available at http://www.nhm.ku.edu/desktopgarp/). All the bioclimatic variables (19 environmental variables) were downloaded from WorldClim website with a resolution of 2.5 arc-min (http://www.worldclim.org/) (Hijmans et al., 2005). The LGM data used in this study were under Community Climate System Model (CCSM; Collins et al., 2006). Because of the environmental layers were of 2.5 arc-min spatial resolution for the “present-day” and LGM periods, the available 30 arc-sec spatial resolution layers of LIG were resampled using ARC GIS 10.0 to obtain same level of resolution. In addition, to reduce the effects of overfitting, ecological niche models (ENMs) based on current climatic data were trained using the methods of Sheppard (2013). Subsequently, only seven variables (BIO4, BIO7, BIO9, BIO11, BIO15, BIO18, BIO19) were used to develop the current distributions for the three oak species (Table S8), while the remaining 12 were discarded because of high autocorrelation. Information on the geographic distribution of related species was based on the 33 populations included in this study, and sites were removed that were separated from each other by <0.1°, so as to reduce the effect of spatial autocorrelation, after that we totally obtained 170 records of the three oak species (50 for *Q. aquifolioides*, 40 for *Q. rehderiana* and 80 for *Q. spinosa*) from Chinese Virtual Herbarium (http://www.cvh.org.cn/cms/). We used the default parameters of MAXENT and included 80% of species records for training and 20% for testing the model and the default convergence threshold (10^−5^) and set the number of maximum iterations to 5000 and the number of replicates to 10. The output format was set to logistic. Models no better than having random predictive ability have an area under the receiver operating characteristic (ROC) curve (AUC) value of 0.5, whereas models with perfect predictive ability have an AUC approximating 1. An AUC score above 0.7 was considered as good model performance (Fielding and Bell, 1997). For GARP, we changed the total run to 100, maximum iterations to 1000 and training proportion to 80. Training data were divided into true training data (for model rule development) and intrinsic testing data (for rule testing intrinsic to GARP processing) (Figure S4). The “best subset” of the total data was generated according to the softomission threshold of 20% and commission threshold of 50%. We predicted the occurrence of each species based on present data and a uniform probability distribution. Finally we used DIVA-GIS v7.5 (Hijmans et al., 2001) and ARC GIS v10.0 to draw the range of suitable distributions.

Furthermore, to assess levels of niche divergence within the three oak species, we used ENMTOOLS v1.3 (Warren et al., 2008) to calculated the niche overlap statistic Schoener's *D* (Schoener, 1968) and standardized Hellinger distance (calculated as *I*). We then used the background similarity test as implemented in ENMTOOLS v1.3 to determine whether the ENMs of different pairs of species are more or less similar than expected from the differences in the environmental backgrounds of the regions where they occur (Warren et al., 2008). The background area should include accessible areas for the three oak species, not just the observed niche or an area tightly delimited by species occurrence (McCormack et al., 2010). The test was run 100 times, and the observed niche overlap was compared to the distribution of overlap values from the runs in each direction to determine whether species' niches were significantly more or less divergent than expected (two-tailed test). Rejection of the null hypothesis demonstrates that observed niche differences are a function of habitat selection and/or availability and may be interpreted as evidence for niche conservatism or niche divergence. Failure to reject the null hypothesis indicates that niche differentiation is explained by variation in the environmental conditions available to each within their respective ranges (Warren et al., 2008, 2010; McCormack et al., 2010).

## Results

### Cp DNA and its sequence polymorphism

**Figure 1:**
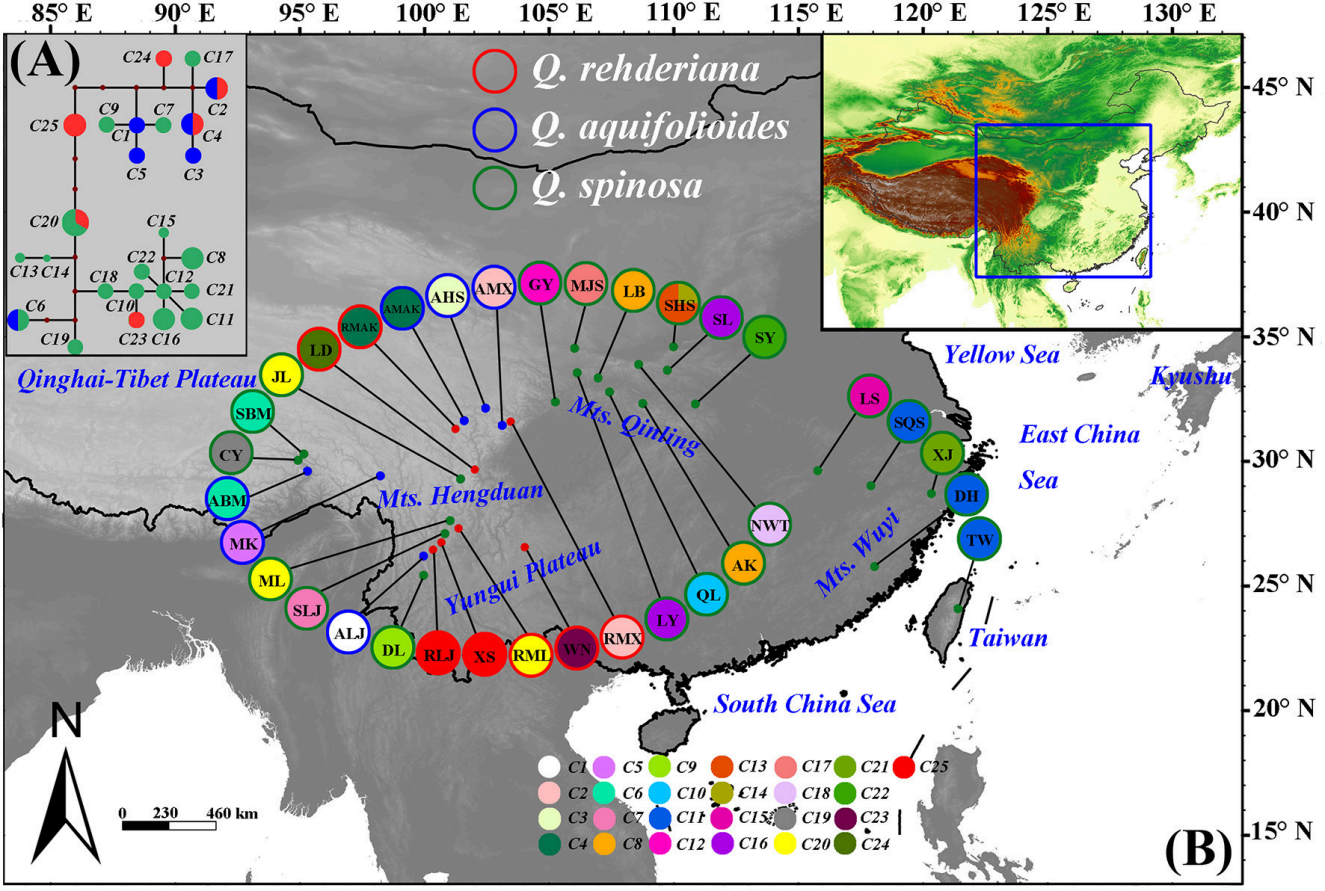
**(A)** Network of the chloroplast (cp) DNA haplotypes detected in three oak species. Different species are denoted by different colors of the circle, each sector of a circle is in proportional to the frequency of each chlorotype. **(B)** Geographic distribution of the chloroplast (cp) DNA haplotypes detected in three oak species. Haplotype frequencies of each population are denoted by the pie charts with population IDs in the circle. Green, blue and red dots indicate the sample locations of *Quercus spinosa, Q. aquifolioides*, and *Q. rehderiana*, respectively.

The concatenated sequences of the three cpDNA fragments were 1785 bp in length, a total of 25 chlorotypes were identified. *Q. aquifolioides* (C1, C3, and C5) and *Q. rehderiana* (C23–C25) had three private haplotypes, respectively, while two haplotypes (C2 and C4) were shared between them. *Q. spinosa* possessed 15 private haplotypes (C7–C19, C21–C22); in addition, it shared C6 with *Q. aquifolioides* and C20 with *Q. rehderiana*, respectively (Figure 1 and Table S2). Estimates of average within-species haplotype diversity was highest in *Q. spinosa* (*H*_d_ = 0.927). Values of *N*_ST_ and *G*_ST_ were both very high in all three species, and in no instance was *N*_ST_ significantly higher than *G*_ST_ (Table 1).

**Table 1:** **Genetic diversity parameters based on cpDNA and ITS sequences in the population groups of the three species**.

**Species**	**Sample size**	***n***	***H*_d_**	**π (× 10^−3^)**	***D***	***F*_S_**	***H*_S_**	***H*_T_**	***N*_ST_**	***G*_ST_**
**cpDNA**										
QA	48	6	0.851	5.82	1.654	1.823	0	1	1.00^a^	1.00^a^
QR	55	6	0.830	8.37	1.965	1.975	0	0.952	1.00^a^	1.00^a^
QS	153	16	0.927	5.96	0.580	2.253	0.024	0.971	0.999	0.975
**ITS**										
QA	24	6	0.797	3.58	0.331	1.127	0.306	0.883	0.848	0.645
QR	28	5	0.762	3.40	0.883	1.245	0	0.857	1.00^a^	1.00^a^
QS	81	10	0.758	4.00	−0.030	1.062	0.103	0.807	0.958	0.872

*n, the number of haplotypes; QA, Quercus aquifolioid; QR, Q. rehderiana; QS, Q. spinosa*.

a *Actually not calculate in the program PERMUTE. Genetic diversity parameters analyzed in this study include haplotype diversities (H_d_), nucleotide diversity (π), Tajima's D and Fu's (F_S_), gene diversity within populations (H_S_), total gene diversity (H_T_), and the coefficients of differentiation (G_ST_ and N_ST_)*.

We obtained a 501-bp fragment of ITS regions, and a total of 14 ribotypes were identified. Among these genotypes, N3 and N5 occurred across the three species, N1 and N4 were unique to *Q. aquifolioides*, while *Q. rehderiana* and *Q. spinosa* possessed two (N13 and N14) and five private ribotypes (N8-N12), respectively (Figure S1A and Table S3). Values of *H*_d_ and *p*_i_ were similar for the three related species. Estimates for average within-population and total ITS diversity were higher in *Q. aquifolioides* (*H*_S_ = 0.306, *H*_T_ = 0.883) than that in the other two species. In *Q. aquifolioides* and *Q. spinosa, N*_ST_ (0.848 and 0.958, respectively) was significantly larger than *G*_ST_ (0.654 and 0.872, respectively) (*p* < 0.05, Table 1), implying the presence of phylogeographic structure.

The AMOVA analyses for cpDNA and ITS data showed that genetic variation mainly occurred among populations within species (77.42 and 76.56%, respectively, Table 2). For nuclear and chloroplastic loci, the Tajima's *D* values were positive in all cases except for the ITS data in *Q. spinosa*; all these values were not statistically significant (Table 1).

**Table 2:** **Analysis of molecular variance (AMOVA) for groups of related species**.

**Source of variation**	***d.f*.**	***SS***	***VC***	***PV* (%)**	**Φ-/*R*–statistic**
**cpDNA**					
Among groups	2	602.42	3.0459	22.56	Φ_SC_ = 0.9997^**^
Among populations within groups	30	2418.56	10.4543	77.42	Φ_ST_ = 0.9998^**^
Within populations	222	0.75	0.0034	0.03	Φ_CT_ = 0.2256^*^
Total	254	3021.73	13.5035		
**ITS**					
Among groups	2	36.32	0.3434	19.09	Φ_SC_ = 0.9462^**^
Among populations within groups	30	167.66	1.3776	76.56	Φ_ST_ = 0.9565^**^
Within populations	99	7.75	0.0783	4.35	Φ_CT_ = 0.1909^*^
Total	131	211.74	1.7994		
**MICROSATELLITE**					
Among groups	2	181.62	0.0898	2.14	*R*_SC_ = 0.3489^**^
Among populations within groups	30	1654.43	1.4310	34.14	*R*_ST_ = 0.3628^**^
Within populations	1185	3165.13	2.6710	63.72	*R*_CT_ = 0.0214^*^
Total	1217	5001.18	4.1918		

Notes: d.f., degrees of freedom; SS, sum of squares; VC, variance components; PV, percentage of variation;

* *p < 0.05*,

** *p < 0.01, 10,000 permutations; Φ_CT_, differentiation among groups within three species; Φ_SC_, differentiation among populations within species; Φ_ST_, differentiation among populations within three species*.

**Figure 2:**
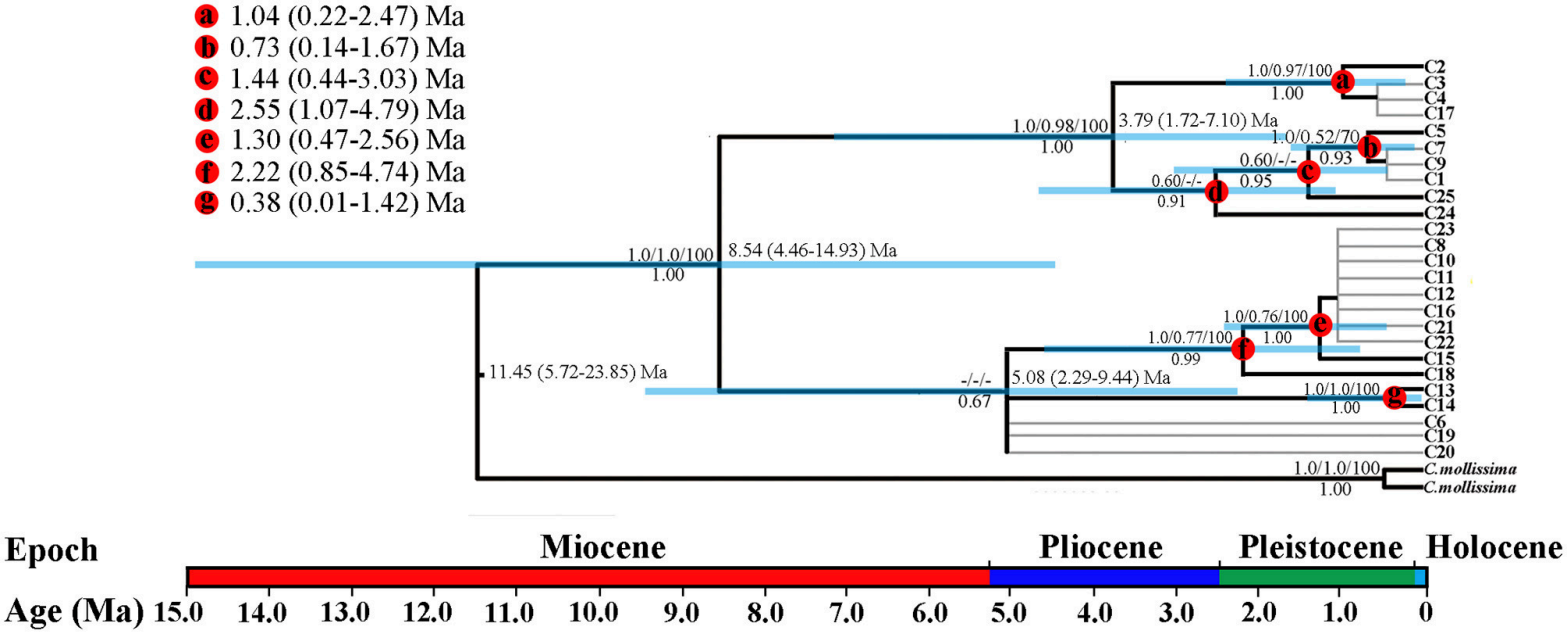
**BEAST-derived chronograms of three oak species based on cpDNA (***psb***A-***trn***H, ***psb***B-***psb***F, and ***matK***) sequences**. Blue bars indicate the 95% highest posterior density (HPD) credibility intervals for node ages (in Myr ago, Ma). Bootstrap values (>50%) based on ML, MP, and BI analysis and posterior probabilities are labeled above and below nodes, respectively. Mean divergence dates and 95% HPDs for major nodes (a–g) are summarized in the upper left figure.

The structure obtained from NETWORK for cpDNA was basically consistent with the topology of the phylogenetic tree (Figures 1A, 2). For cpDNA data, C20, C12, and C25 took central positions, and might be more ancient. Furthermore, almost all populations harbored one private haplotype. When referred to the ITS subsets (Figure S1A), N5 and N7 took central positions in the network with a higher proportion of individuals (16.54 and 9.02%, respectively). N3 was found to be the predominant ribotypes, and was mainly distributed from the central China outward to the adjacent EH-HM regions with the highest proportion of individuals (32.33%).

### Molecular dating based on cpDNA data

The BEAST-derived chronograms of oak species based on three cpDNA sequences suggested that the divergence could date back to 11.45 million years ago (Ma) (95% highest posterior density, HPD: 5.72–23.85 Ma, PP = 1.00), indicating a Mid-Miocene split between the species and the outgroup. And the coalescent time of all chlorotypes within species (*c*. 8.54 Ma, 95% HPD: 4.46–14.93 Ma, PP = 1.00) indicated that the oak species diverged in Late Miocene (Figure 2). In addition, strong differentiation occurred within taxon during the periods of Quaternary climatic oscillations.

### Nuclear microsatellite diversity and population structure

The null allele test indicated a lower frequency of null allele at each of the 12 loci than the threshold frequency (□ = 0.15) across the 33 populations, and there was no evidence for LD. After Bonferroni correction, significant deviation from HWE induced by homozygote excess was detected in all 12 loci when all samples were treated as a single population within species. However, there were no HWE deviations within each population after Bonferroni correction.

Genetic diversity indices were summarized for each loci and species (Table 3 and Table S4 in). Amplification of all 12 loci in 609 individuals revealed a total of 211 alleles with a range of 7–33 alleles per locus, and the mean *H*_T_ and *H*_S_ were 0.699 and 0.673 with a range of 0.279–0.905 and 0.278–0.891, respectively. Population differentiation was significant for all 12 loci (*p* < 0.05, Table S4) with the mean *F*_ST_ value for multilocus estimate being 0.371 (range: 0.120–0.543). As for the standardized genetic differentiation, due to the high *H*_T_ and variable nSSR makers used in this study, *G*'_ST_ was higher than *F*_ST_ across all loci (Table S4), which was in line with previous studies [Meirmans and Hedrick, 2010; also see review by Edelaar and Björklund (2011)]. Within species, mean *A*_S_ and *H*_E_ were similar, ranging from 12.5 to 13.8 and from 0.642 to 0.694, respectively. On average, *Q. rehderiana* had the highest *F*_IS_ value whereas *Q. aquifolioides* had the highest *H*_O_ value.

**Table 3:** **Diversity measures in the related species**.

**Locus**	**QS**				**QA**				**QR**			
	***A*_S_**	***H*_O_**	***H*_E_**	***F*_IS_**	***A*_S_**	***H*_O_**	***H*_E_**	***F*_IS_**	***A*_S_**	***H*_O_**	***H*_E_**	***F*_IS_**
3A05	11.7	0.454	0.761	0.402	11.8	0.479	0.828	0.422	14.2	0.543	0.778	0.303
3D15	13.7	0.334	0.787	0.576	13.4	0.538	0.731	0.265	15.2	0.264	0.620	0.574
1P10	11.1	0.217	0.667	0.675	11.5	0.445	0.799	0.444	12.8	0.150	0.752	0.801
2P24	6.8	0.114	0.296	0.614	9.6	0.067	0.066	−0.027	8.3	0.136	0.348	0.611
ZAG9	15.4	0.506	0.810	0.376	18.3	0.571	0.842	0.322	17.5	0.400	0.753	0.470
ZAG11	12.2	0.186	0.572	0.676	10.9	0.395	0.661	0.403	12.1	0.293	0.702	0.583
ZAG15	14.0	0.474	0.668	0.291	11.0	0.597	0.797	0.252	14.0	0.421	0.789	0.467
ZAG20	17.8	0.546	0.861	0.366	19.5	0.706	0.927	0.239	20.0	0.486	0.883	0.451
ZAG30	23.3	0.554	0.898	0.383	22.2	0.588	0.907	0.352	24.3	0.457	0.812	0.438
ZAG46	9.0	0.114	0.623	0.817	9.7	0.353	0.679	0.481	9.7	0.179	0.729	0.756
MSQ4	3.7	0.200	0.260	0.232	3.8	0.210	0.252	0.167	4.4	0.250	0.322	0.225
MSQ13	11.7	0.237	0.500	0.526	12.5	0.261	0.667	0.610	12.7	0.350	0.839	0.584
Mean	12.5	0.328	0.642	0.495	12.9	0.434	0.679	0.328	13.8	0.327	0.694	0.522

*The parameters presented below include allelic richness (AS) after rarefaction (rarefaction size = 119), observed (HO) and expected heterozygosity (HE), inbreeding coefficient (FIS) per locus. Notes: QR, Quercus rehderiana; QS, Q. spinosa; QA, Q. aquifolioides*.

**Figure 3:**
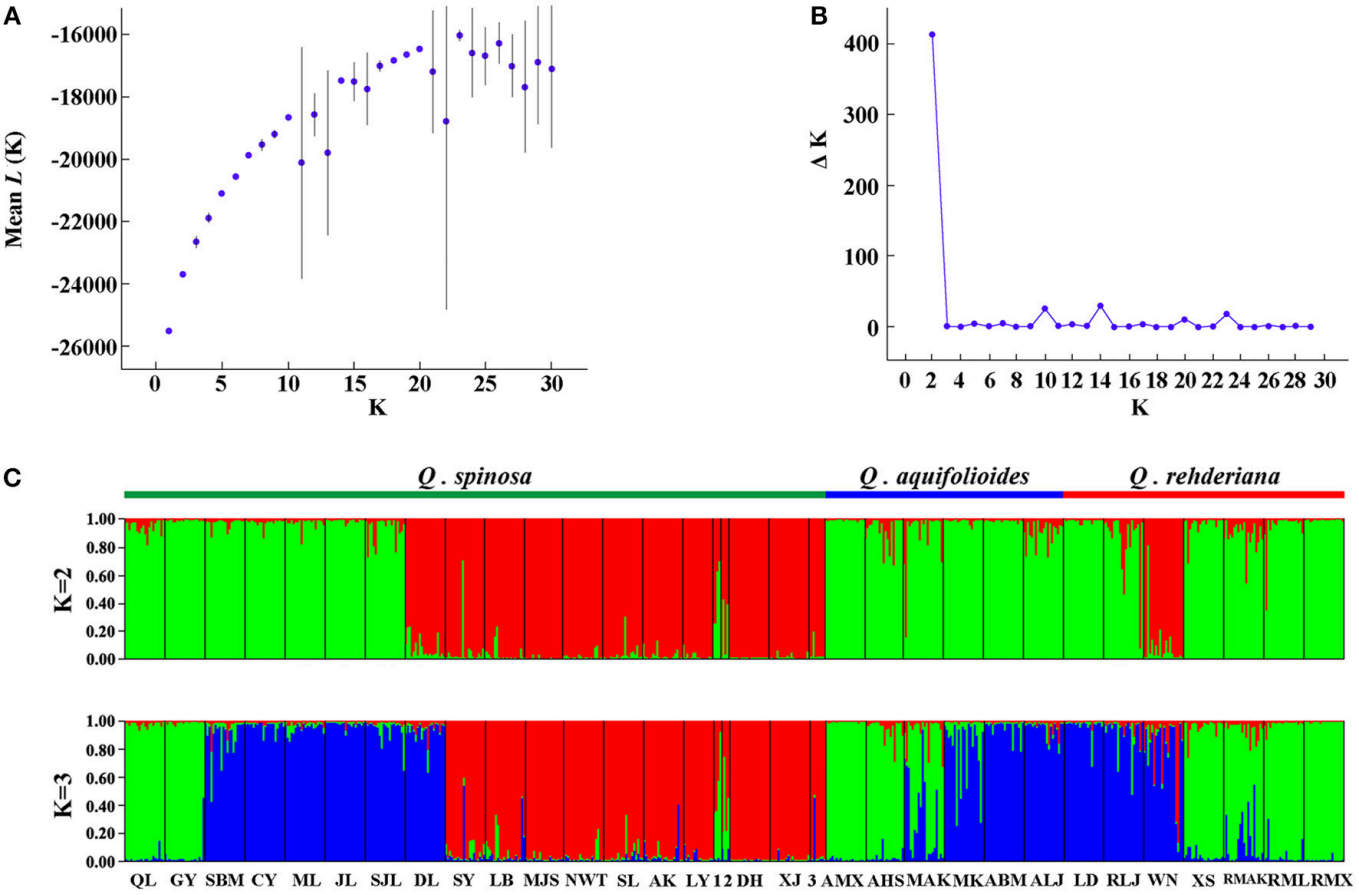
**Bayesian inference analysis of microsatellite data for determining the most likely number of clusters (***K***) for the three species**. The distribution of the likelihood *L*(*K*) values **(A)** and Δ*K* values **(B)** are presented for *K* = 1–30 (10 replicates). STRUCTURE plots **(C)** are presented for best *K* = 2 and *K* = 3, respectively (1 = SHS; 2 = LS; 3 = SQS).

The nSSR-derived AMOVA demonstrated significant genetic differentiation (*R*_ST_ = 0.3628, *p* < 0.01), and the majority (63.72%) of the variation partitioned within populations (Table 2). There was no obvious IBD for any of the three species (Figure S2). For the Bayesian analysis of population structure, the most likely number of genetic clusters was estimated at 2 (Figure 3). Populations of *Q. spinosa*, including one population (DL) from Yunnan Province, those from the east of Mts. Hengduan (except GY and QL) and one population of *Q. rehderiana* (WN), formed one group; the other group comprised all populations of *Q. aquifolioides*, six populations of *Q. rehderiana* (except WN), and the remaining populations of *Q. spinosa* from Mts. Hengduan and Yungui Platea. (Figure S3A). Populations occurred in the EH-HM region were further subdivided into three sub-clusters, which was in line with the delimitation of three oak species occurred there (Figure S3), however, the low Δ*K* value implied, indeed, the genetic structure of these three sub-clusters had some similarities.

The Wilcoxon test under the SMM and TPM models failed to detect any recent genetic bottleneck in any of the populations, and all populations showed an L-sharp allele distribution when ruling out two populations (Table S5, SHS and LS with smaller population sizes). According to the 2MOD analysis, the most likely model of population history which led to the current population structure was gene flow-drift model rather than pure drift model (*P* = 1.0, Bayesian factor = 100,000).

### Gene flow within the three oak species

Our study revealed interesting patterns of historical and contemporary gene flow among the four clusters. Almost all the historical gene flow of the related pairs were symmetrical with slight differences, however, there were significant asymmetrical contemporary gene flow in related pairs except for that within *Q. spinosa* (Table 4). It should be pointed out that we used the program IMa2 (Hey, 2010) to estimate the gene flow between related pairs in the beginning, however, we couldn't obtain ideal results due to its disequilibrium results of parameter settings in our study and it's slow computational speed.

**Table 4:** **Rates of historical and contemporary gene flows per generation among the four clusters as estimated using microsatellite data with the programs MIGRATE and BAYESASS, respectively**.

	**QS1**	**QS2**	**QA**	**QR**
**MIGRATE–N**				
QS1	–	0.6079 (0.5270–0.6983)	2.1570 (1.9531–2.3798)	3.1905 (2.2930–3.817)
QS2	0.7630 (0.6626–0.8751)	–	2.4913 (2.2684–2.7326)	2.4196 (2.1993–2.6609)
QA	1.9150 (1.7332–2.1124)	2.4234 (2.2267–2.6352)	–	0.9751 (0.8532–1.1118)
QR	3.1754 (2.9192–3.4512)	1.9470 (1.7803–2.1272)	0.7573 (0.6565–0.8701)	–
**BAYESASS**				
QS1	–	0.0024 (0–0.0071)	**0.0656** (0.0423–0.0889)	**0.0027** (0–0.0080)
QS2	0.0016 (0–0.0047)	–	**0.0017** (0–0.0048)	**0.0017** (0–0.0050)
QA	**0.0046** (0–0.128)	**0.0059** (0–0.0143)	–	**0.0553** (0.0030–0.1076)
QR	**0.0976** (0.0723–0.1229)	**0.0393** (0.0103–0.0683)	**0.0028** (0–0.0083)	–

*Notes: QA, Quercus aquifolioid; QR, Q. rehderiana; QS, Q. spinosa. Values in bold indicate asymmetric gene flow between related pairs. Ninety-Five percent confidence intervals are presented in parentheses*.

### Evolutionary dynamics and changes in effective population size

In the DIYABC analysis, the posterior probabilities for scenarios 2 was 0.5539 [95% confidence interval (CI) 0.5139–0.5940], much higher than other eight scenarios. The median values of the effective population sizes of QS1, QS2, QA, QR and NA were 3.75 × 10^5^, 1.19 × 10^4^, 2.57 × 10^5^, 5.22 × 10^5^, and 1.68 × 10^5^, respectively. The estimated median time of divergence between QS1, QS2, and QR (t1), and the divergence time between QA and the ancestor of QS1 and QS2 (t2) were 3.24 × 10^4^ and 7.58 × 10^4^ generations ago, respectively. Assuming the generation time to be 150 years, the divergence times of t1 and t2 corresponded to 4.86 Ma and 11.37 Ma, respectively. The estimated median mutation rates and the proportion of multiple step mutations in the generalized stepwise model of microsatellites were estimated to be 1.75 × 10^−5^ and 0.564, respectively (Table 5).

**Table 5:** **Posterior median estimate and 95% highest posterior density interval (HPDI) for demographic parameters in scenarios 2 based on the nuclear multilocus microsatellite data for three oak species**.

**Parameters**	**QS1 ^a^**	**QS2 ^b^**	**QA ^c^**	**QR ^d^**	**NA ^e^**	**t1 (generations)**	**t2 (generations)**	**μ**	***P***
Median	3.75E+05	1.19E+04	2.57E+05	5.22E+05	1.68E+04	3.24E+04	7.58E+04	1.75E−05	0.564
Lower_bound	1.10E+05	2.67E+03	7.34E+04	1.87E+05	1.21E+03	1.04E+04	2.24E+04	2.43E−06	0.333
Upper_bound	8.73E+05	1.19E+05	7.70E+05	9.32E+05	1.72E+05	3.94E+04	1.39E+05	9.01E−03	0.846

a−e *The current population sizes of QS1, QS2, QA, and QR were denoted as NQS1, NQS2, NQA, and NQR, respectively, while NA represents the population size of the ancestral lineage at time t2. t1: the divergence time between QR, QS1, and QS2. t2: the time of ancient divergence. μ: mutation rate (per generation per locus)*.

Our nuclear microsatellite data for the three closely related oak species were best fitted with the contraction-expansion model [posterior probability = 0.8114 (95% CI: 0.7933–0.8295), 0.7247 (95% CI: 0.7093–0.8401) and 0.6583 (95% CI: 0.6440–0.6725) for the *Q. spinosa, Q. aquifolioides* and *Q. rehderiana*, respectively]. As indicated by the contraction-expansion model, the point estimates of the current population sizes for the *Q. spinosa, Q. aquifolioides* and *Q. rehderiana* were 5.72 × 10^5^, 5.43 × 10^5^, and 5.72 × 10^5^, respectively, the population sizes between current and ancient were 7.41 × 10^2^, 2.00 × 10^3^, and 1.56 × 10^3^, respectively, and the ancient population sizes were 5.31 × 10^5^, 4.84 × 10^5^, and 4.60 × 10^5^, respectively. The contraction time was 3.17 × 10^4^, 3.22 × 10^4^, and 3.21 × 10^4^ generations ago for the *Q. spinosa, Q. aquifolioides* and *Q. rehderiana*, respectively (*c*. 4.80, 4.83, and 4.82 Ma if a generation length of 150 years is assumed), and the expansion time was 3.03 × 10^3^, 3.67 × 10^3^, and 3.53 × 10^3^ generations ago for the *Q. spinosa, Q. aquifolioides*, and *Q. rehderiana*, respectively (*c*. 0.45, 0.55, and 0.53 Ma if a generation length of 150 years is assumed) (Table S9).

### Ecological niche modeling

**Figure 4:**
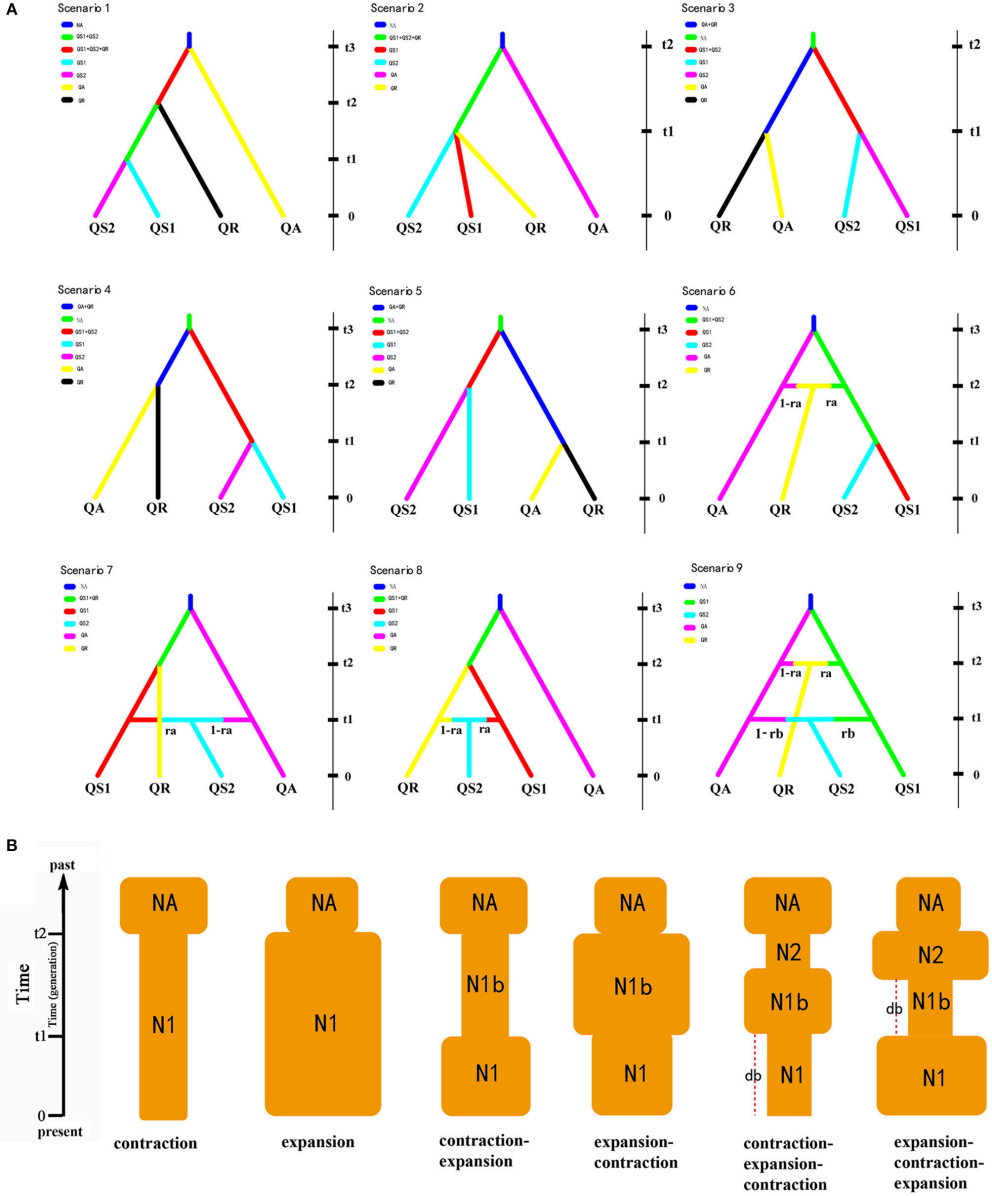
**(A)** The nine scenarios of the population history of three oak species in DIYABC V2.04. QS1 and QS2 represent two groups of *Quercus spinosa*; QA and QR represent *Q. aquifolioides* and *Q. rehderiana*, respectively. The current population sizes of QS1, QS2, QA, and QR were denoted as NQS1, NQS2, NQA, and NQR, respectively, while NA represents the population size of the ancestral lineage at time t3 (or t2). t1–t3, divergence times for the depicted event. **(B)** Schematic representation of six demographic models of changes in population size tested within three oak species (*Quercus spinosa, Q. aquifolioides*, and *Q. rehderiana*) using DIY-ABC. NA, ancestral population size; N1, current population size; N1b and N2, populations sizes between NA and N1; db, duration of bottleneck.

**Figure 5:**
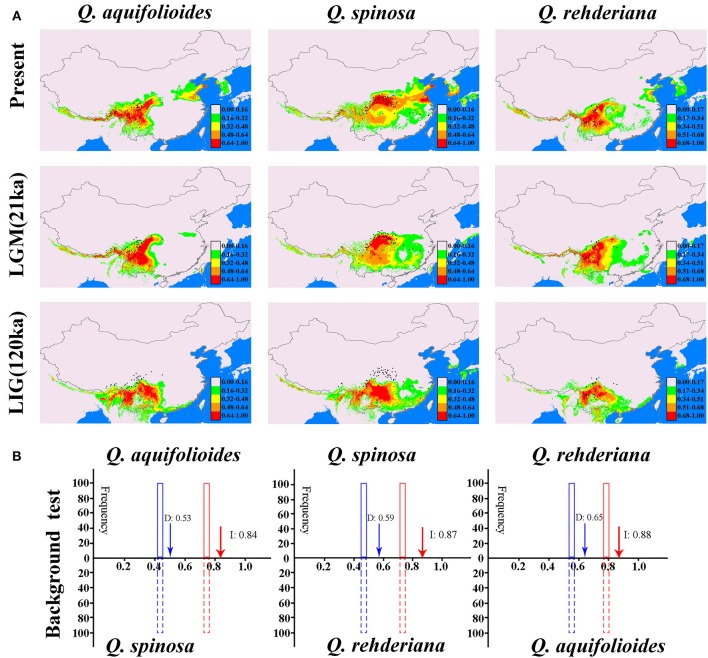
**(A)** Predicted distributions of *Quercus aquifolioides, Q. spinosa*, and *Q. rehderiana* based on ecological niche modeling using MAXENT, black dots indicate extant occurrence points. **(B)** The background tests between different pairs of species. Null distributions are shown by dotted blue bars for *D* and solid red bars for *I*. The x-axis indicates values of *I* and *D*, and y-axis indicates number of randomizations. Red and blue arrows indicate values in actual MAXENT runs, respectively.

There were similar change tendencies of suitable distributions of the three oak species obtained by MAXENT and GARP (Figure 5A and Figure S4). All models had high predictive ability (AUC > 0.9). In addition, the projected present-day distribution is consistent with collection records (Figure 4A). The ENMs suggested the three oak species continued their expansions during the LGM. Since the LGM, however, except for the *Q. spinosa* has distinct expansion, there have been little change in the predicted spatial distributions of the other two species despite climate warming (Figure 4A).

For background tests, the observed niche overlap values for *I* and *D* were significantly higher (*p* < 0.001) than the predicted scores under the null hypothesis both for *Q. aquifolioides* vs. *Q. spinosa, Q. spinosa* vs. *Q. rehderiana*, and *Q. rehderiana* vs. *Q. aquifolioides* (Figure 4B), indicating that the niches of all three species were very different and more conserved than expected based on the environments available for occupation by each. Additionally, the values of *I* and *D* greater than 0.5 in all three pairs of species, which indicated that the niches of all the species pairs were slightly overlapped (Figure 5B).

## Discussion

### High genetic diversity and strong genetic differentiation

The three species investigated in our study are characterized by long generation times, wind pollination, and seed dispersal through animals and gravity; all these characteristics are associated with high genetic diversity and differentiation (Hamrick et al., 1979, 1992). Our study did observe high genetic diversities for all three species based on both cpDNA and ITS datasets (*H*_T_ = 0.971–1 and 0.807–0.883, respectively), and the estimated differentiation values (*G*_ST_) were larger than the mean genetic differentiation calculated for maternally inherited plastid markers in white oaks of Europe (0.975–1 vs. 0.848) (Petit et al., 2002), implying the existence of strong barriers to gene flow. The high mountains, gorges, and complex paleo-drainage basins in EH-HM region have served as effective barriers to seed and/or pollen dispersal and impacted genetic diversification as well as differentiation of organisms, which has also been reported in other phylogeographic studies (e.g., Li et al., 2013; Liu et al., 2013). In addition, the low ability of seeds is also an important factor contributing to high population differentiation. Most of the *Fagus* seeds simply drop to the ground near their parent trees and a few may roll down on steep terrain, sometimes animals (e.g., jays or squirrels) may also carry some seeds short distances (Gómez, 2003; Xiao et al., 2009). This may also be the case for the three oak species, although their seed dispersal modes are still to be studied in detail.

Our results of AMOVA (Table 2) demonstrated that both cpDNA and ITS marker systems are strongly differentiated among populations but almost homogenous within populations. When combined with the general lack of IBD, The island-like population genetic structure of three oak species in accordance with their highly fragmented habitats located in the EH-HM region, and largely driven by historical effects of genetic drift rather than currently limited gene flow alone (Hutchison and Templeton, 1999). In fact, similar genetic structure have also been detected in other species in this region, such as *Garrulax elliotii* from the eastern Himalayas (Qu et al., 2011) and four herbs from HHM region (Luo et al., 2015).

### Demographic history and ranges expansion of the three oak species

The uplift of the QTP since the Cenozoic had a significant impact on species differentiation and genetic structure (Qiu et al., 2011). Some studies have shown that the main uplifting of QTP occurred in 10 Ma (Mulch and Chamberlain, 2006), reaching peak elevation already shortly before the Late Pliocene (Sun et al., 2011). Our molecular dating by cpDNA (Figure 2) and SSR data both fell into the late Miocene. The split between the related oak species and *C. mollissima* (Figure 2) might due to the enhanced monsoon climate have triggered differentiation between these species after the uplifting of the QTP at 15–13 Ma (An et al., 2001). Initial differentiation within taxa at 8.54 Ma (95% HPD: 4.46–14.93 Ma) may be explained by expanding arid areas in Asian inland, the effect of East Asian monsoon before 9–8 Ma, and the tempestuous uplift of the QTP during this period (An et al., 2001). This may have resulted in lots of small fragmented habitats with different microclimate, thereby impacting the direction of natural selection (Sobel et al., 2010). Furthermore, our ABC simulation indicated the divergence time of *Q. rehderiana* was 4.86 Ma (95%HPD: 3.36–5.91 Ma), implying a Pliocene split. Likewise, molecular dating of cpDNA data revealed dramatic divergence since 5.08 Ma (95%HPD: 2.29–9.44 Ma, Figure 2). Our divergence time estimation coincides with a period of intense uplift of the Hengduan Mountain massif (Sun et al., 2011; Favre et al., 2015), suggesting that the split could similarly have occurred during the colonization of the newly available terrain by these oak species. In fact, an association between Pliocene uplift of the QTP and time of intraspecific and/or interspecific divergence has previously been noted in this region (e.g., Li et al., 2013; Sun et al., 2014). It is plausible, that species had fragmented habitats caused by the QTP uplifting during Pliocene may have fostered both intraspecific and interspecific divergence on a large scale in the region.

However, the divergence time presented here should be treated with caution because we utilized the substitution rates of *Q. variabilis*, which is a deciduous tree and has shorter generation time than the three species, might be underestimate the divergence time. Another nuanced caveat here is some inherent problems of SSR data, for instance, homoplasy, trending to underestimate divergence time on large time scales (Selkoe and Toonen, 2006). Moreover, the assumption of no gene flow in DIYABC also leads to the underestimation of the divergence time between species (Leaché et al., 2013). However, many Miocene fossils belonging to group *Ilex* were discovered in the QTP could not be classified as any extant species (Zhou, 1993), it seems likely that the divergence time of the three oak species fell into the Miocene.

Our ABC demographic analysis detected a contraction-expansion model for all the three oak species, their effective population sizes decreased within the past *c*. 4.80 Ma and increased around *c*. 0.5 Ma. Although the EH-HM region became colder during the Pliocene due to the extensive uplift of the QTP (Shi et al., 1999), which facilitated the growing of three oak species. However, other alpine species (e.g., spruce, Sun et al., 2014) in this region could also adapt this climate, occupying some of the niches originally belonged to oaks in the present study and enlarged their ranges via successful competition. Based on the process above, the three closely oak species might decrease their population sizes. Although the temperature on the QTP increased at the end of the largest glaciation (ca. 1.2–0.6 Ma), the relatively cold climate may have continued until the late Ionian stage (0.3–0.126 Ma) (Shi et al., 1998). Thus, because these sclerophyllous oaks could adapt cold habitats (Zhou et al., 1994), it is feasible that they expanded their ranges and increased their population sizes during this period. The fact that a moderately cold climate has prevailed on the EH-HM region as the LIG will have provided opportunities for these three species to have continued their ranges expansion. Indeed, our ENM analyses also suggested that all the three species had experienced ranges expansion from LIG (0.14–0.12 Ma) to LGM, which was similar to other alpine plants reported in this region, e.g., *P. likiangensis* (Li et al., 2013) and *T. wallichiana* (Liu et al., 2013). It seems therefore, that trees such as mentioned above, which occur in cold environments nowadays, may have exhibited different range dynamics during past climatic oscillations relative to species associated with warmer environments.

### Genetic admixture and asymmetric contemporary gene flow

Although these three oak species were morphologically differentiated, they were genetically admixed in the present study due to incomplete lineage sorting and interspecific hybridization. Our results indicated that the haplotypes N2, N3, and N5 shared among species are ancient and have disjunctive distribution (Figure S1B), and shared haplotypes C2, C4, C6, C20, and N6 between sympatric species (Figure 1 and Figure S1B) as expected for incomplete lineage sorting (Maddison and Knowles, 2006) and introgressive hybridization, respectively.

Generally, numerous studies have revealed shared DNA polymorphisms between closely related species or species complex (e.g., Du et al., 2009; Li et al., 2012; Simeone et al., 2016). This situation can be divided into two categories: firstly, retention of ancestral polymorphisms which caused by incomplete lineage sorting during and following speciation (Heckman et al., 2007; Wilyard et al., 2009); secondly, introgression or introgressive hybridization which caused by genetic exchange after secondary contact between previously geographically separated species (Liston et al., 1999; Gay et al., 2007). Incomplete lineage sorting occurs when lineages fail to coalesce in the ancestral population of species (Pamilo and Nei, 1988; Maddison, 1997; Degnan and Salter, 2005). Therefore, the probability of incomplete lineage sorting depends on both the effective population size (*Ne*) in the ancestral population of species, which determines the rate of coalescence of lineages, and the time between successive speciation events (Hudson, 1990; Degnan and Salter, 2005). While the introgression is mostly detected in zones of sympatry/parapatry between two or more species, in other words, it occurs in co-distributed populations belong to different species [Ortego et al., 2015; also see review by Abbott et al. (2013) and references therein].

Our results of 2MOD strongly supported the gene flow-drift model, indicating that interspecific hybridization significantly contributed to the observed genetic admixture between the three species. Despite these three oaks have weak boundaries and close phylogenetic relationships (Figures 1A, 2), the possibility that the shared ancestral polymorphism can't be completely rejected. In this regard, the real cause of genetic admixture within these oak species deserves further study.

Our Bayesian cluster analysis (Figure 3C) indicated that populations from EH-HM region could be considered as a single cluster disregarding the species boundaries. One plausible explanation is that, despite existing genetic barriers in the EH-HM region, due to changes of their distribution ranges that give rise to sympatric distribution as well as existing incomplete reproductive isolation within oak species, gene flow (Table 4) among them will blur species boundaries. Similar situations were reported in other studies (Du et al., 2011; Ma et al., 2014). Populations in the east of Mts. Qinling (Figure S3B) could be another cluster due to analogous environments resulting in phenotypic convergence or fixed similar alleles during population expansions (Excoffier and Ray, 2008).

Our results indicated there were asymmetric contemporary gene flow for five related pairs whereas the historical gene flow for all pairs seemed symmetric (Table 4). A possible explanation for the asymmetric contemporary gene flow is the species richness in the EH-HM region. Generally, at the present time, *Q. spinosa* occurs in low-altitude regions, while *Q. aquifolioides* grows in high-altitude regions; both are common keystone species in local forests, however, *Q. rehderiana* occupies moderate-altitude areas with scattered distribution, such distribution patterns usually lead to stronger gene flow from core populations to peripheral ones as suggested by Alleaume-Benharira et al. (2006). In addition, human activities in mid-/low-altitude regions during the past decades would more significantly affect the population sizes of *Q. rehderiana*, giving rise to the current model of gene flow.

In general, gene flow is a vital factor for population structure over time, it may reduce local adaptation by homogenizing populations found in differing environments or by spreading detrimental alleles across populations, and it also serves to introduce potentially adaptive alleles to populations, and increases genetic diversity (Sexton et al., 2011; Epps and Keyghobadi, 2015; Welt et al., 2015). Previous study have suggested that contemporary and historical gene flow were generally low and similar among populations occurred in highly fragmented habitats (Chiucchi and Gibbs, 2010), and if genetic structure was weak or admixture, it might due to strong historical gene flow (Epps et al., 2013). Indeed, our results (Table 4) indicated the historical and contemporary gene flow among these oak species were generally low, however, whether the genetic admixture is due to historical gene flow rather than contemporary ones, we need further investigation because oak species themselves have weak boundaries.

## Conclusions

Our analyses of the three related species reveal similar demographic history according to ENMs and ABC analyses. The neutral genetic markers did not depict the profile of differentiation entirely due to introgression or incomplete lineage sorting. Geological and climatic changes since Miocene have markedly affected the observed patterns of genetic variation and divergence among and within species. Our study indicates that a combination of orographic and bioclimatic analyses can yield deep insights into the diversification and evolutionary history of species in the EH-HM and adjacent regions.

## Author contributions

GZ and ZL designed the research; LF and JY collected samples; LF, QZ, and YZ performed experiments; LF and ZQ analyzed data; LF and ZQ prepared figures and tables; LF, ZQ, and ZL wrote the manuscript; and LF, QZ, ZQ, JY, YZ, ZL, and GZ revised the manuscript.

### Conflict of interest statement

The authors declare that the research was conducted in the absence of any commercial or financial relationships that could be construed as a potential conflict of interest.

## Supplementary material

Figure S1 **(A)** Network of the ITS haplotypes detected in three oak species. Different species are denoted by different colors of the circle, each sector of a circle is in proportional to the frequency of each haplotype. **(B)** Geographic distribution of the ITS haplotypes detected in three oak species. Haplotype frequencies of each population are denoted by the pie charts with population IDs in the circle. Green, blue and red dots indicate the sample locations of *Quercus spinosa, Q. aquifolioides*, and *Q. rehderiana*, respectively.

Figure S2 **Mantel tests between microsatellite-based genetic distances and geographic distances. (A)** *Quercus spinosa*; **(B)** *Q. aquifolioides*; **(C)** *Q. rehderiana*.

Figure S3 **Bayesian inference analysis of microsatellite data for determining the most likely number of clusters (K) for the three lineages of oak species occurred in the EH-HM region**. The distribution of the likelihood L(K) values **(A)** and ΔK values **(B)** are presented for K = 1–15 (10 replicates). STRUCTURE plots **(C)** are presented for best K = 3 (QS1: populations of the *Quercus spinosa* with green in Figure 3C; QA: *Q*. *aquifolioides*; QR: *Q*. *rehderiana*).

Figure S4 **Ecological niche models predicted distributions of the three species using GARP during three periods**. Different colors corresponded to different fitting indices with low in blue and high in red for the current, LGM and LIG distribution.

Table S1 **Sampling details and haplotypes detected in each population of the three related species**.

Table S2 **The variable sites in aligned cpDNA sequences of ***psbA-trnH, psbB-psbF***, and ***matK*** that yielded 25 chlorotypes (C1–C25) recorded across the three species from 34 populations**.

Table S3 **The variable sites in aligned nrDNA sequences detected in the ***ITS4-ITS5*** region of the three related species from 33 populations, which identified 14 haplotypes (N1–N14)**.

Table S4 **Genetic diversity parameters estimated at 12 microsatellite loci in 33 populations**.

Table S5 **Bottleneck analysis for 33 populations of the three related species**.

Table S6 **The nine scenarios for four lineages**.

Table S7 **Prior distributions for model parameters used in model comparisons**.

Table S8 **The 19 bioclimatic variables used in the full set of variables, with √ indicating the variables used in the customized sets for ***Q. spinosa*** (QS), ***Q. rehderiana*** (QR), and ***Q. aquifolioides*** (QA); the variables used in the reduced set**.

Table S9 **Posterior median estimate and 95% highest posterior density interval (HPDI) for demographic parameters of an contraction-expansion model based on the nuclear multilocus microsatellite data of three oak species, respectively**.

Table S10 **The SSR dataset of these three oak species in this study**.

Note S1 **Description of the oak species and their habitats in our study**.

Note S2 **Methodological details for sequences and microsatellite genotyping**.
