# Supplementary figures and images for: Genetic Structure and Evolutionary History of Three Alpine Sclerophyllous Oaks in East Himalaya-Hengduan Mountains and Adjacent Regions
Source: Front Plant Sci. 2016 Nov 11;7:1688. doi: 10.3389/fpls.2016.01688 (PMC5104984; doi:10.3389/fpls.2016.01688)

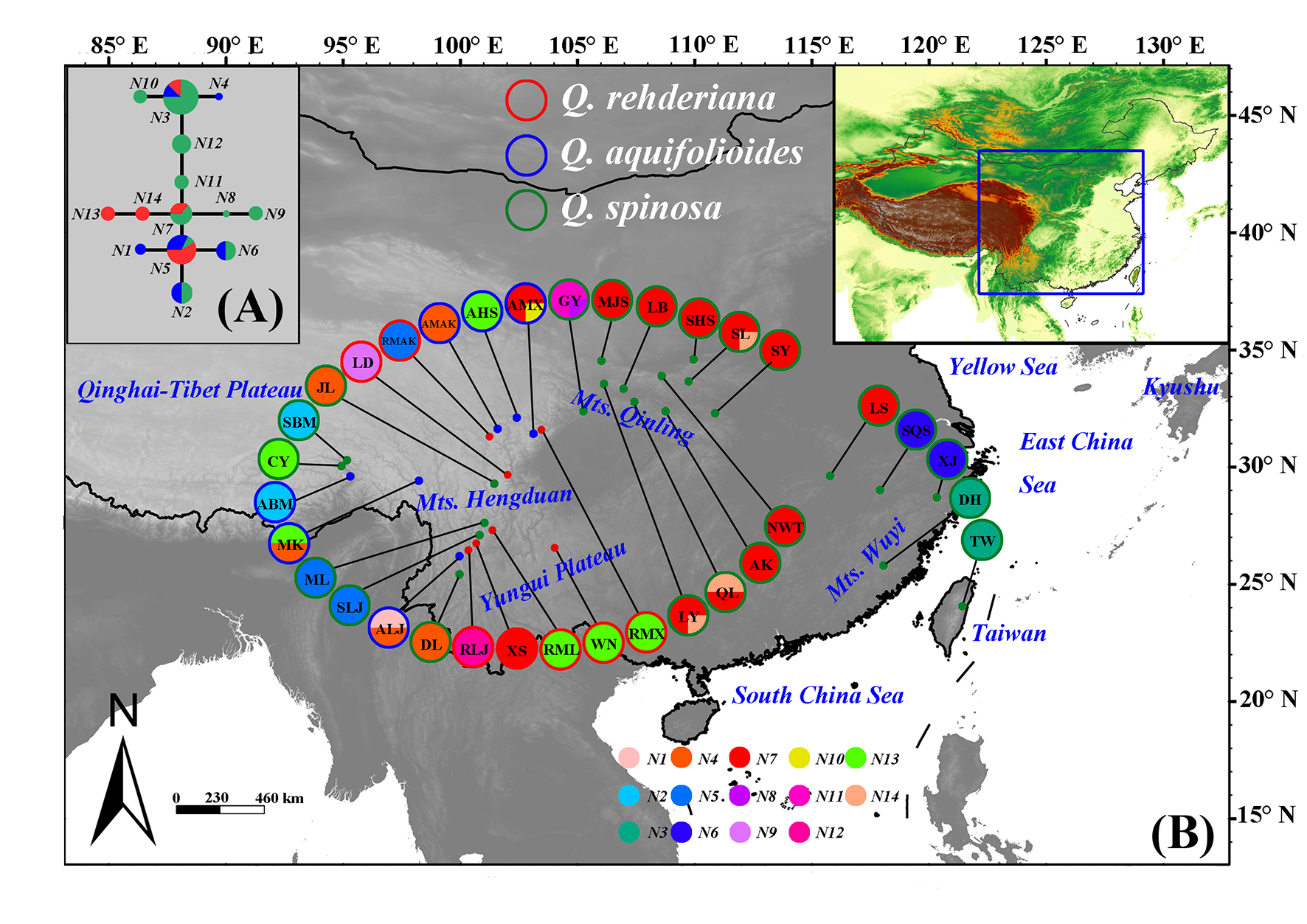

Supplement: Figure S1 — (A) Network of the ITS haplotypes detected in three oak species. Different species are denoted by different colors of the circle, each sector of a circle is in proportional to the frequency of each haplotype. (B) Geographic distribution of the ITS haplotypes detected in three oak species. Haplotype frequencies of each population are denoted by the pie charts with population IDs in the circle. Green, blue and red dots indicate the sample locations of Quercus spinosa, Q. aquifolioides, and Q. rehderiana, respectively. [file Image1.TIF]

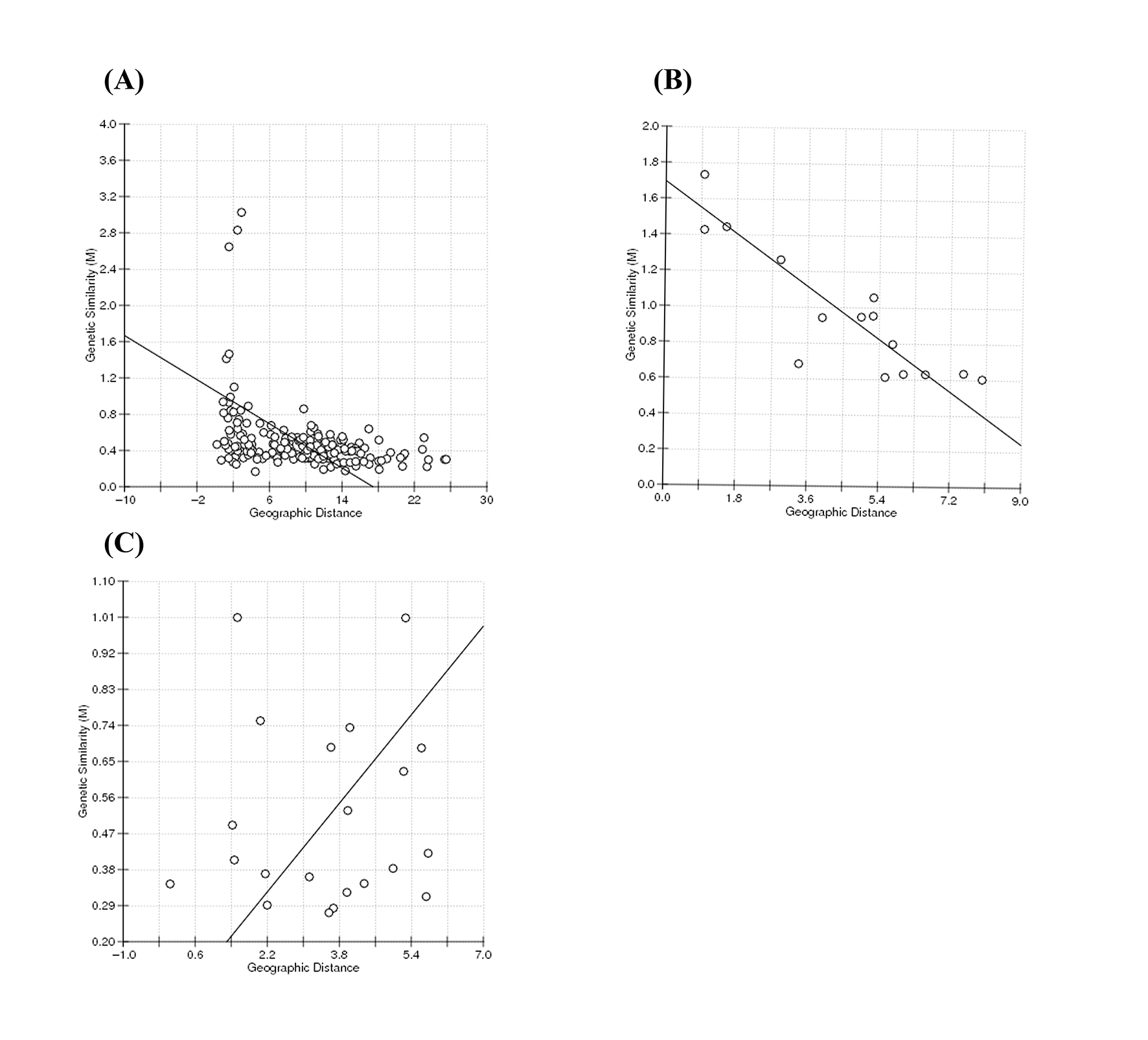

Supplement: Figure S2 — Mantel tests between microsatellite-based genetic distances and geographic distances. (A) Quercus spinosa; (B) Q. aquifolioides; (C) Q. rehderiana. [file Image2.TIF]

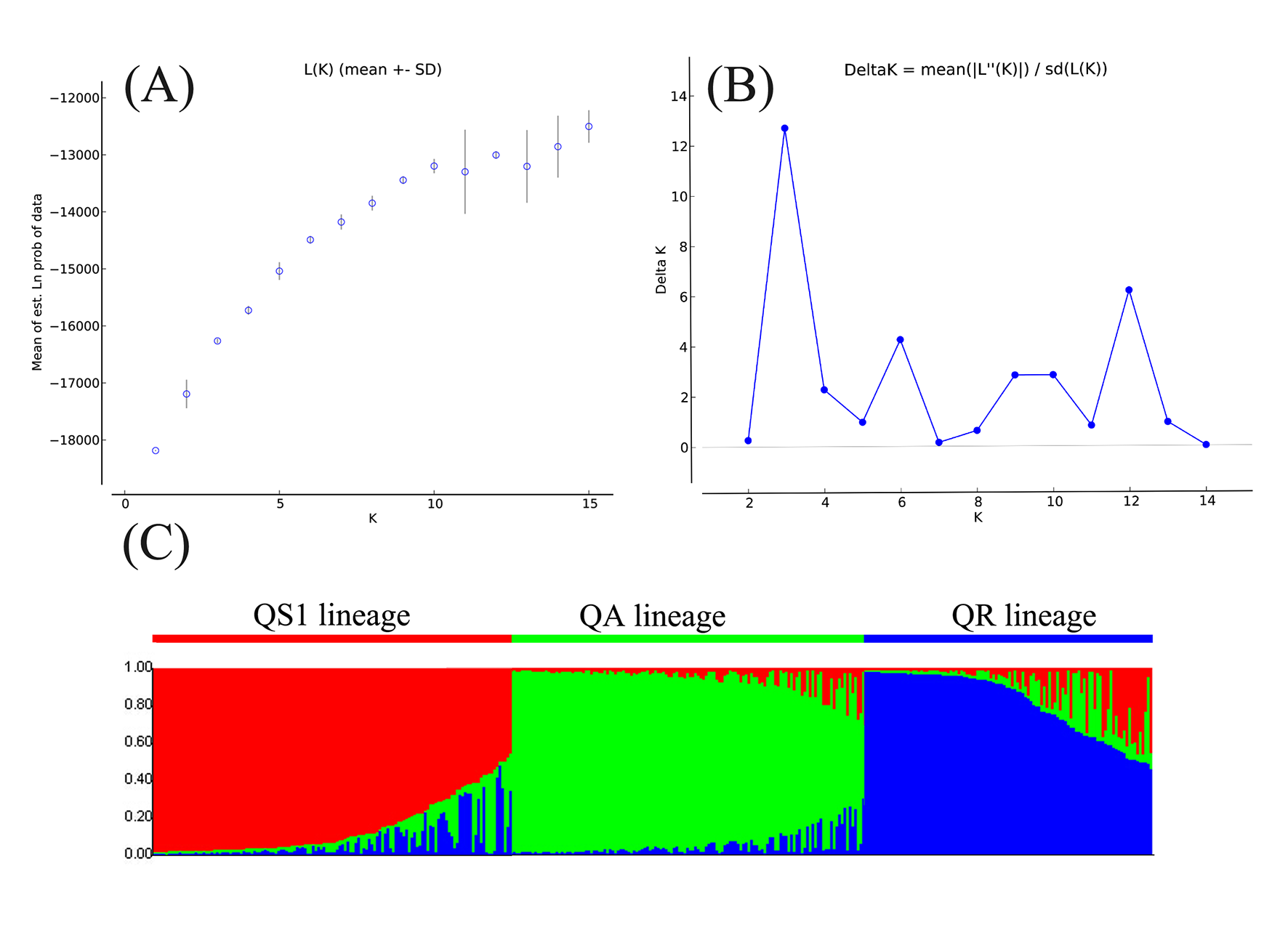

Supplement: Figure S3 — Bayesian inference analysis of microsatellite data for determining the most likely number of clusters (K) for the three lineages of oak species occurred in the EH-HM region. The distribution of the likelihood L(K) values (A) and ΔK values (B) are presented for K = 1–15 (10 replicates). STRUCTURE plots (C) are presented for best K = 3 (QS1: populations of the Quercus spinosa with green in Figure 3C; QA: Q. aquifolioides; QR: Q. rehderiana). [file Image3.TIF]

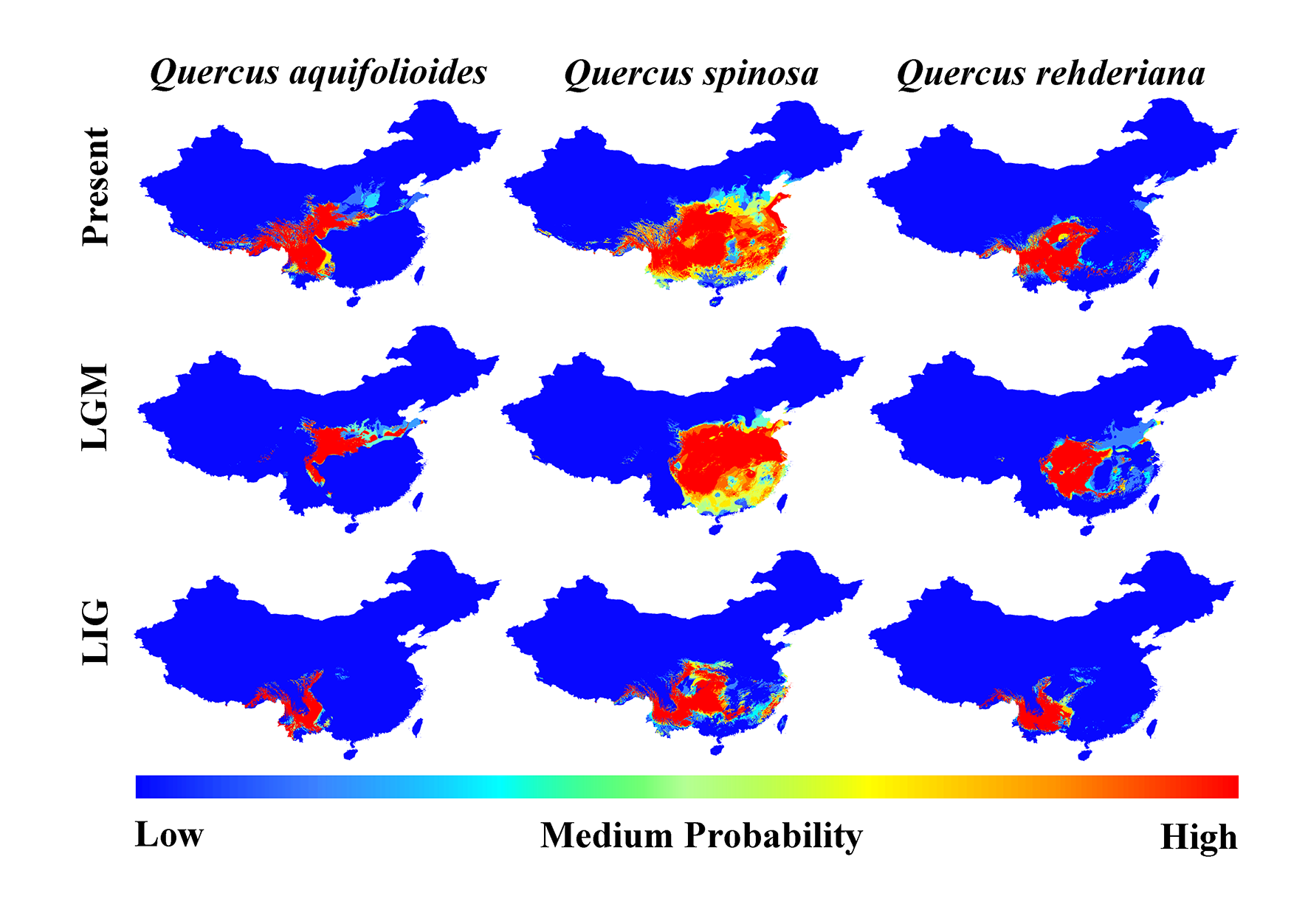

Supplement: Figure S4 — Ecological niche models predicted distributions of the three species using GARP during three periods. Different colors corresponded to different fitting indices with low in blue and high in red for the current, LGM and LIG distribution. [file Image4.TIF]
