# Supplementary material for: Genetic Structure and Evolutionary History of Three Alpine Sclerophyllous Oaks in East Himalaya-Hengduan Mountains and Adjacent Regions
Source: Front Plant Sci. 2016 Nov 11;7:1688. doi: 10.3389/fpls.2016.01688 (PMC5104984; doi:10.3389/fpls.2016.01688)
Supplement: Table S1 — Sampling details and haplotypes detected in each population of the three related species. [file Table1.DOCX]

**Table S1** Sampling details and haplotypes detected in each population of the three related species

| Pop ID | Species | Location | Latitude (°N) | Longitude (°E) | Altitude (m) | Sample size | Chlorotype | Ribotype |
| --- | --- | --- | --- | --- | --- | --- | --- | --- |
| QL | *Q. spinosa* | Qinling, Shaanxi | 32.74 | 107.52 | 1,700 | 20^a^/8^b^/4^c^ | C10 | N3,N10 |
| SGY | *Q. spinosa* | Guangyuan, Sichuan | 32.24 | 105.38 | 735 | 20/8/4 | C12 | N8,N9 |
| ML | *Q. spinosa* | Muli, Sichuan | 27.56 | 101.16 | 2,088 | 20/8/4 | C20 | N7 |
| JL | *Q. spinosa* | Jiulong, Sichuan | 29.24 | 101.56 | 3,764 | 20/8/4 | C20 | N2 |
| SY | *Q. spinosa* | Shiyan, Hubei | 32.23 | 111.01 | 1,519 | 20/8/4 | C22 | N3 |
| SLJ | *Q. spinosa* | Lijiang, Yunnan | 26.54 | 100.15 | 2,658 | 20/8/4 | C7 | N7 |
| LB | *Q. spinosa* | Liuba, Shaanxi | 33.28 | 107.09 | 1,740 | 20/8/4 | C8 | N3 |
| SDL | *Q. spinosa* | Dali, Yunnan | 25.39 | 100.07 | 1,960 | 20/8/4 | C9 | N2 |
| CY | *Q. spinosa* | Cayu, Xizang | 30.05 | 95.04 | 2,066 | 20/8/4 | C19 | N5 |
| MJS | *Q. spinosa* | Tianshui, Gansu | 34.46 | 106.12 | 1,330 | 19/8/4 | C17 | N3 |
| NWT | *Q. spinosa* | Xi'an, Shaanxi | 33.86 | 108.68 | 1,200 | 20/8/4 | C18 | N3 |
| SL | *Q. spinosa* | Shangluo, Shaanxi | 33.53 | 109.89 | 1,310 | 20/8/4 | C16 | N3,N10 |
| AK | *Q. spinosa* | Ankang, Shaanxi | 32.33 | 108.9 | 1,660 | 20/8/4 | C8 | N3 |
| LY | *Q. spinosa* | Hanzhong, Shaanxi | 33.51 | 106.22 | 1,680 | 15/8/4 | C16 | N3,N10 |
| SHS | *Q. spinosa* | Huashan, Shaanxi | 34.53 | 110.1 | 2,300 | 4/4/4 | C13,C14 | N3 |
| LS | *Q. spinosa* | Jiujiang, Jiangxi | 29.55 | 115.96 | 1,980 | 4/4/4 | C15 | N3 |
| DH | *Q. spinosa* | Dehua, Fujiang | 25.7 | 118.19 | 1,789 | 20/8/4 | C11 | N11 |
| XJ | *Q. spinosa* | Xianju, Zhejiang | 28.62 | 120.51 | 1,752 | 20/8/4 | C21 | N12 |
| SQS | *Q. spinosa* | Shangrao, Jiangxi | 28.93 | 118.08 | 1,665 | 8/8/4 | C11 | N12 |
| SBM | *Q. spinosa* | Bomi, Xizang | 30.16 | 95.22 | 3,107 | 20/8/4 | C6 | N6 |
| TW | *Q. spinosa* | Hualien, Taiwan | 23.99 | 121.61 | 2,410 | -/1/1 | C11 | N11 |
| AMX | *Q. aquifolioides* | Maoxian, Sichuan | 31.37 | 103.24 | 1,954 | 20/8/4 | C2 | N3,N4 |
| AHS | *Q. aquifolioides* | Heishui, Sichuan | 32.05 | 102.55 | 2,965 | 19/8/4 | C3 | N5 |
| MAK | *Q. aquifolioides* | Maerkang, Sichuan | 31.54 | 101.71 | 2,770 | 20/8/4 | C4 | N2 |
| MK | *Q. aquifolioides* | Mangkang, Xizang | 29.41 | 98.36 | 4,049 | 20/8/4 | C5 | N2,N5 |
| ABM | *Q. aquifolioides* | Bomi, Xizang | 29.52 | 95.45 | 2,728 | 20/8/4 | C6 | N6 |
| ALJ | *Q. aquifolioides* | Lijiang, Yunnan | 26.53 | 100.14 | 2,658 | 20/8/4 | C1 | N1,N2 |
| LD | *Q. rehderiana* | Luding, Sichuan | 29.54 | 102.12 | 1,789 | 20/8/4 | C24 | N13 |
| PLJ | *Q. rehderiana* | Lijiang, Yunnan | 26.52 | 100.13 | 2,658 | 20/8/4 | C25 | N14 |
| WN | *Q. rehderiana* | Weining, Guizhou | 26.46 | 104.12 | 2,136 | 20/8/4 | C23 | N5 |
| RMX | *Q. rehderiana* | Maoxian, Sichuan | 31.38 | 103.25 | 1,954 | 20/8/4 | C2 | N5 |
| RML | *Q. rehderiana* | Muli, Sichuan | 27.55 | 101.18 | 2,100 | 20/8/4 | C20 | N5 |
| RLJ | *Q. rehderiana* | Lijiang, Yunnan | 26.56 | 100.15 | 2,700 | 20/8/4 | C25 | N3 |
| RMAK | *Q. rehderiana* | Maerkang, Sichuan | 31.55 | 101.72 | 2,560 | 20/8/4 | C4 | N7 |

Note: ^a^ sample sizes in the analysis of microsatellite; ^b^ sample sizes in the analysis of cpDNA; ^c^ sample sizes in the analysis of ITS.
