# Supplementary material for: Genetic Structure and Evolutionary History of Three Alpine Sclerophyllous Oaks in East Himalaya-Hengduan Mountains and Adjacent Regions
Source: Front Plant Sci. 2016 Nov 11;7:1688. doi: 10.3389/fpls.2016.01688 (PMC5104984; doi:10.3389/fpls.2016.01688)
Supplement: Table S2 — The variable sites in aligned cpDNA sequences of psbA-trnH, psbB-psbF, and matK that yielded 25 chlorotypes (C1–C25) recorded across the three species from 34 populations. [file Table2.DOCX]

**Table S2** The variable sites in aligned cpDNA sequences of *psbA-trnH*, *psbB-psbF* and *matK* that yielded 25 chlorotypes (C1-C25) recorded across the three species from 34 populations

| Dataset | Variable nucleotide sites | | | | | | | | | | | | | | | | | | | | | | | | | | | | | | |  |
| --- | --- | --- | --- | --- | --- | --- | --- | --- | --- | --- | --- | --- | --- | --- | --- | --- | --- | --- | --- | --- | --- | --- | --- | --- | --- | --- | --- | --- | --- | --- | --- | --- |
| cpDNA | Chlorotype | 0  0  3 | 0  0  9 | 10  ︱  12 | 0  1  4 | 0  1  6 | 0  2  2 | 0  2  4 | 0  2  5 | 0  2  7 | 0  3  3 | 0  3  5 | 37  ︱  40 | 0  7  1 | 0  7  2 | 1  0  2 | 1  2  7 | 1  4  0 | 151  ︱  154 | 1  9  7 | 228  ︱  241 | 242  ︱  280 | 2  8  1 | 288  ︱  291 | 292  ︱  297 | 3  2  6 | 3  3  2 | 3  9  2 | 4  2  7 | 432  ︱  439 | 4  4  2 |  |
| *psbA-trnH* | C1 | A | G | ▲ | C | C | A | A | A | A | A | T | ■ | A | T | T | A | T | ♠ | G | ● | - | G | ○ | - | C | A | G | C | ✪ | A |  |
|  | C2 | G | G | ▲ | C | C | A | A | A | A | A | T | ■ | A | T | G | A | T | ♥ | G | ● | - | G | - | - | C | G | G | C | ✪ | A |  |
|  | C3 | G | G | ▲ | C | C | A | A | A | A | A | T | ■ | A | T | G | A | T | ♣ | G | ● | - | G | - | - | C | A | G | C | ✪ | A |  |
|  | C4 | G | G | ▲ | C | C | A | A | A | A | A | T | ■ | A | T | G | A | T | ♣ | G | ● | - | G | - | - | C | A | G | C | ✪ | A |  |
|  | C5 | A | G | ▲ | C | C | A | A | A | A | A | T | ■ | A | T | T | A | T | - | G | ● | 🟏 | G | ○ | - | C | A | G | C | ✪ | A |  |
|  | C6 | A | A | ♦ | A | T | T | T | T | T | G | G | □ | A | T | G | C | T | ♣ | G | ● | - | G | 🞈 | ★ | C | A | G | T | ✪ | A |  |
|  | C7 | A | G | ▲ | C | C | A | A | A | A | A | T | ■ | A | T | T | A | C | ♠ | G | - | - | - | ○ | - | C | A | G | C | ✪ | A |  |
|  | C8 | A | A | ♦ | A | T | T | T | T | T | G | G | □ | T | A | G | A | T | ♣ | A | ● | - | G | - | - | C | A | G | C | - | A |  |
|  | C9 | A | G | ▲ | C | C | A | A | A | A | A | T | ■ | A | T | T | A | T | ♠ | G | ● | - | G | ○ | - | C | A | G | C | ✪ | A |  |
|  | C10 | A | A | ♦ | A | T | T | T | T | T | G | G | □ | A | T | G | A | T | ○ | A | ● | - | G | - | - | C | A | G | C | ✪ | A |  |
|  | C11 | A | A | ♦ | A | T | T | T | T | T | G | G | □ | A | T | G | A | T | ♣ | A | ● | - | G | - | - | C | A | G | C | ✪ | A |  |
|  | C12 | A | A | ♦ | A | T | T | T | T | T | G | G | □ | A | T | G | A | T | ♣ | A | ● | - | G | - | - | C | A | G | C | ✪ | A |  |
|  | C13 | A | A | ♦ | A | T | T | T | T | T | G | G | □ | A | T | G | A | T | ♣ | G | ● | - | G | - | - | C | G | G | C | ✪ | A |  |
|  | C14 | A | A | ♦ | A | T | T | T | T | T | G | G | □ | A | T | G | A | T | ♣ | G | ● | - | G | - | - | C | G | G | C | ✪ | A |  |
|  | C15 | A | A | ♦ | A | T | T | T | T | T | G | G | □ | A | T | G | A | T | ♣ | A | ● | - | G | - | - | C | A | T | C | - | A |  |
|  | C16 | A | A | ♦ | A | T | T | T | T | T | G | G | □ | A | T | G | A | T | ♣ | A | ● | - | G | - | - | C | A | G | C | ✪ | A |  |
|  | C17 | G | G | ▲ | C | C | A | A | A | A | A | T | ■ | A | T | G | A | T | ♥ | G | ● | - | G | - | - | C | A | G | C | ✪ | A |  |
|  | C18 | A | A | ♦ | A | T | T | T | T | T | G | G | □ | A | T | G | A | T | ○ | A | ● | - | G | - | - | C | A | G | C | ✪ | A |  |
|  | C19 | A | A | ♦ | A | T | T | T | T | T | G | G | □ | A | T | G | A | T | ○ | G | ● | - | G | 🞊 | 🟅 | C | A | G | C | ✪ | A |  |
|  | C20 | A | A | ♦ | A | T | T | T | T | T | G | G | □ | A | T | T | A | T | ♠ | G | ● | - | G | ○ | - | C | A | G | C | ✪ | A |  |
|  | C21 | A | A | ♦ | A | T | T | T | T | T | G | G | □ | A | T | G | A | T | ♣ | A | ● | - | G | - | - | C | A | G | C | ✪ | A |  |
|  | C22 | A | A | ♦ | A | T | T | T | T | T | G | G | □ | A | T | G | A | T | ♣ | A | ● | - | G | - | - | C | A | G | C | ✪ | A |  |
|  | C23 | A | A | ♦ | A | T | T | T | T | T | G | G | □ | A | T | G | A | T | ○ | A | ● | - | G | - | - | C | A | G | C | ✪ | G |  |
|  | C24 | A | G | ▲ | C | C | A | A | A | A | A | T | ■ | A | T | T | A | T | ♠ | G | ● | - | G | ○ | - | T | A | G | C | ✪ | A |  |
|  | C25 | A | G | ▲ | C | C | A | A | A | G | A | T | ▼ | A | T | T | A | T | ♠ | G | ● | - | G | ○ | - | C | A | G | C | ✪ | A |  |
| cpDNA | Chlorotype | 0  1  2 | 0  5  7 | 0  7  4 | 113  ︱  117 | 1  1  8 | 120  ︱  122 | 2  5  3 | 3  3  7 | 3  8  7 | 3  9  3 | 4  4  3 | 6  0  2 | 7  2  2 | cpDNA | 0  0  7 | 0  2  5 | 0  2  6 | 0  4  9 | 0  7  9 | 0  9  9 | 1  7  1 | 2  0  2 | 2  0  9 | 3  4  1 | 3  4  5 | 3  7  0 | 3  8  1 | 4  1  5 | 5  0  4 | 5  5  3 | 5  6  3 |
| *psbB-psbF* | C1 | G | G | T | 🟑 | A | - | C | A | A | C | C | A | T | *matK* | A | A | T | T | G | T | G | G | C | C | C | C | T | T | T | G | A |
|  | C2 | A | G | T | 🟑 | A | - | C | A | A | T | C | A | C |  | A | A | T | C | G | T | G | G | C | T | C | G | T | T | T | G | C |
|  | C3 | A | G | T | 🟑 | A | - | C | A | A | T | C | A | C |  | A | A | T | T | G | T | G | G | C | T | C | G | T | G | T | G | C |
|  | C4 | A | G | T | 🟑 | A | - | C | A | A | T | C | A | C |  | A | A | T | T | G | T | G | G | C | T | C | G | T | T | T | G | C |
|  | C5 | G | G | T | 🟑 | C | - | C | A | A | C | C | A | T |  | A | A | T | T | G | T | G | G | C | C | C | C | T | T | T | G | A |
|  | C6 | G | G | T | 🟑 | A | - | C | A | A | T | C | A | C |  | A | A | T | T | G | T | G | G | C | C | C | G | T | T | T | G | A |
|  | C7 | G | G | T | 🟑 | A | - | C | A | A | C | C | A | T |  | A | A | T | T | G | T | G | G | C | C | C | C | T | T | T | G | A |
|  | C8 | G | G | T | 🟑 | A | - | T | A | A | T | C | A | C |  | C | A | T | T | G | T | G | G | C | C | T | G | T | T | T | G | A |
|  | C9 | G | G | T | 🟑 | A | - | C | A | A | C | C | A | T |  | A | A | T | T | G | T | G | G | C | C | C | C | C | T | T | G | A |
|  | C10 | G | G | T | 🟑 | A | - | T | A | A | T | C | A | C |  | C | A | T | T | G | T | G | G | C | C | T | G | T | T | T | G | A |
|  | C11 | G | G | T | 🟑 | A | - | T | G | A | T | C | A | C |  | C | A | T | T | G | T | G | G | C | C | T | G | T | T | T | G | A |
|  | C12 | G | G | T | 🟑 | A | - | T | A | A | T | C | A | C |  | C | A | T | T | G | T | G | G | C | C | T | G | T | T | T | G | A |
|  | C13 | G | G | T | 🟑 | A | - | C | A | A | T | C | A | C |  | A | A | T | C | G | T | G | G | C | T | C | G | T | T | T | G | C |
|  | C14 | G | G | T | 🟑 | A | - | T | A | A | T | C | A | C |  | A | A | T | C | G | T | G | G | C | T | C | G | T | T | T | G | C |
|  | C15 | G | G | T | 🟑 | A | - | T | A | A | T | C | A | C |  | C | A | T | T | G | C | G | G | C | C | T | G | T | T | T | G | A |
|  | C16 | G | G | T | 🟑 | A | - | T | A | A | T | C | G | C |  | C | A | T | T | G | T | G | G | C | C | T | G | T | T | T | G | A |
|  | C17 | A | G | T | - | A | - | C | A | A | T | C | A | C |  | A | G | T | T | G | T | G | G | C | T | C | G | T | T | T | G | C |
|  | C18 | G | G | T | 🟑 | A | - | T | A | A | T | C | A | C |  | A | A | T | T | G | T | G | G | C | C | C | G | T | T | T | G | A |
|  | C19 | G | G | T | 🟑 | A | - | C | A | A | T | C | A | C |  | A | A | T | T | G | T | G | G | C | C | C | G | T | T | T | G | A |
|  | C20 | G | G | T | 🟑 | A | - | C | A | A | C | C | A | T |  | A | A | T | T | G | T | G | G | C | C | C | C | T | T | T | G | A |
|  | C21 | G | G | T | 🟑 | A | - | T | A | G | T | C | A | C |  | C | A | T | T | G | T | G | G | C | C | T | G | T | T | T | G | A |
|  | C22 | G | G | T | 🟑 | A | - | T | A | A | T | C | A | C |  | C | A | T | T | G | T | G | A | C | C | T | G | T | T | T | G | A |
|  | C23 | G | G | T | 🟑 | A | - | T | A | A | T | T | A | C |  | C | A | T | T | G | T | G | G | C | C | T | G | T | T | T | G | A |
|  | C24 | G | T | C | 🟑 | A | 🟁 | C | A | A | T | C | A | T |  | A | A | G | T | T | T | A | G | T | C | C | G | T | T | C | T | A |
|  | C25 | G | G | T | 🟑 | A | - | C | A | A | T | C | A | T |  | A | A | T | T | G | T | G | G | C | C | C | C | T | T | T | G | A |

Note: Dashes indicate missing nucleotides. ▲TCA ♦ATC ■GATT □TGAC ▼TATT ♠T--- ♥TT-- ♣TTT- ○TTTT ●TTTTTATCGGTGAA 🟏GTTTTTATTTTTTTATTTATATATGTTTTTATCGGTGAA 🞈TTTC 🞊TTTA ★TTTTTC 🟅TTTTTA ✪AGTGGGGG 🟑GATTT 🟁TAA
