# Supplementary material for: Genetic Structure and Evolutionary History of Three Alpine Sclerophyllous Oaks in East Himalaya-Hengduan Mountains and Adjacent Regions
Source: Front Plant Sci. 2016 Nov 11;7:1688. doi: 10.3389/fpls.2016.01688 (PMC5104984; doi:10.3389/fpls.2016.01688)
Supplement: Table S3 — The variable sites in aligned nrDNA sequences detected in the ITS4-ITS5 region of the three related species from 33 populations, which identified 14 haplotypes (N1–N14). [file Table3.DOCX]

**Table S3** The variable sites in aligned nrDNA sequences detected in the *ITS4-ITS5* region of the three related species from 33 populations, which identified 14 haplotypes (N1-N14)

| Haplotype | Variable nucleotide positions | | | | | | | | | | | | | | | |
| --- | --- | --- | --- | --- | --- | --- | --- | --- | --- | --- | --- | --- | --- | --- | --- | --- |
|  | 6 | 11 | 12 | 14 | 30 | 62 | 111 | 124 | 128 | 129 | 182 | 188 | 368 | 373 | 406 | 425 |
| N1 | A | - | - | C | G | C | G | A | A | G | - | A | G | G | A | A |
| N2 | A | - | - | C | G | C | G | A | A | G | - | A | G | G | G | G |
| N3 | A | G | G | C | G | C | G | G | A | G | - | G | A | G | G | A |
| N4 | A | G | G | C | G | C | G | G | A | G | G | G | A | G | G | A |
| N5 | A | - | - | C | G | C | G | A | A | G | - | A | G | G | G | A |
| N6 | A | - | - | T | G | C | G | A | A | G | - | A | G | G | G | A |
| N7 | A | - | - | C | G | C | G | A | A | G | - | A | A | G | G | A |
| N8 | G | - | - | C | G | T | G | A | A | G | - | A | A | G | G | A |
| N9 | G | - | - | C | A | T | A | A | A | G | - | A | A | G | G | A |
| N10 | A | G | G | C | G | C | G | G | G | G | - | G | A | G | G | A |
| N11 | A | - | - | C | G | C | G | A | A | G | - | G | A | G | G | A |
| N12 | A | G | G | C | G | C | G | A | A | G | - | G | A | G | G | A |
| N13 | A | - | - | C | G | C | G | A | A | A | - | A | A | A | G | A |
| N14 | A | - | - | C | G | C | G | A | A | G | - | A | A | A | G | A |

Note: Dashes indicate missing nucleotides.
