# Supplementary material for: Genetic Structure and Evolutionary History of Three Alpine Sclerophyllous Oaks in East Himalaya-Hengduan Mountains and Adjacent Regions
Source: Front Plant Sci. 2016 Nov 11;7:1688. doi: 10.3389/fpls.2016.01688 (PMC5104984; doi:10.3389/fpls.2016.01688)
Supplement: Table S4 — Genetic diversity parameters estimated at 12 microsatellite loci in 33 populations. [file Table4.DOCX]

**Table S4** Genetic diversity parameters estimated at 12 microsatellite loci in 33 populations

| Locus | *A_O_* | *H_O_* | *H_E_* | *H_S_* | *H_T_* | *F_ST_* | *G'_ST_* |
| --- | --- | --- | --- | --- | --- | --- | --- |
| 3A05 | 18 | 0.478 | 0.829 | 0.790 | 0.850 | 0.443 | 0.810 |
| 3D15 | 19 | 0.356 | 0.767 | 0.713 | 0.751 | 0.543 | 0.848 |
| 1P10 | 17 | 0.246 | 0.722 | 0.741 | 0.752 | 0.440 | 0.740 |
| 2P24 | 14 | 0.110 | 0.267 | 0.237 | 0.243 | 0.439 | 0.497 |
| ZAG9 | 22 | 0.494 | 0.830 | 0.802 | 0.829 | 0.276 | 0.685 |
| ZAG11 | 14 | 0.250 | 0.625 | 0.646 | 0.652 | 0.314 | 0.523 |
| ZAG15 | 16 | 0.486 | 0.756 | 0.752 | 0.788 | 0.364 | 0.690 |
| ZAG20 | 22 | 0.565 | 0.892 | 0.891 | 0.905 | 0.225 | 0.726 |
| ZAG30 | 33 | 0.539 | 0.900 | 0.873 | 0.898 | 0.251 | 0.761 |
| ZAG46 | 12 | 0.176 | 0.675 | 0.679 | 0.697 | 0.534 | 0.775 |
| MSQ4 | 7 | 0.212 | 0.272 | 0.278 | 0.279 | 0.120 | 0.121 |
| MSQ13 | 17 | 0.268 | 0.681 | 0.670 | 0.744 | 0.499 | 0.738 |
| *Mean* | *17.6* | *0.348* | *0.685* | *0.673* | *0.699* | *0.371* | *0.660* |

Notes: Observed number of alleles (*A*_O_); observed heterozygosity over all populations (*H*_O_); expected heterozygosity over all populations (*H*_E_); gene diversity within populations (*H*_S_); overall gene diversity (*H*_T_); among-population differentiation (*F*_ST_) and standardized genetic differentiation (*G’*_ST_).
