# Supplementary material for: Genetic Structure and Evolutionary History of Three Alpine Sclerophyllous Oaks in East Himalaya-Hengduan Mountains and Adjacent Regions
Source: Front Plant Sci. 2016 Nov 11;7:1688. doi: 10.3389/fpls.2016.01688 (PMC5104984; doi:10.3389/fpls.2016.01688)
Supplement: Table S5 — Bottleneck analysis for 33 populations of the three related species. [file Table5.DOCX]

**Table S5** Bottleneck analysis for 33 populations of the three related species

| Pop ID | Wilcoxon’s sign-rank test | | Mode-shift test (distribution shape) |  | | Pop ID | Wilcoxon’s sign-rank test | | | Mode-shift test (distribution shape) | |
| --- | --- | --- | --- | --- | --- | --- | --- | --- | --- | --- | --- |
|  | TPM | SMM |  |  | |  | TPM | SMM |  | |  |
| QS |  |  |  |  | | DH | 0.922 | 0.922 | L-shaped | |  |
| QL | 0.913 | 0.913 | L-shaped ^a^ | | XJ | | 0.385 | 0.461 | L-shaped | |  |
| SGY | 0.711 | 0.768 | L-shaped |  | | SQS | 0.991 | 0.993 | L-shaped | |  |
| SBM | 0.973 | 0.992 | L-shaped |  | | QA |  |  |  | |  |
| CY | 1 | 1 | L-shaped |  | | AMX | 0.74 | 0.84 | L-shaped | |  |
| ML | 0.993 | 0.996 | L-shaped |  | | AHS | 0.99 | 0.992 | L-shaped | |  |
| JL | 0.999 | 1 | L-shaped |  | | MAK | 0.949 | 0.973 | L-shaped | |  |
| SLJ | 0.999 | 0.999 | L-shaped |  | | MK | 0.999 | 1 | L-shaped | |  |
| SY | 0.976 | 0.99 | L-shaped |  | | ABM | 0.988 | 0.991 | L-shaped | |  |
| LB | 0.998 | 0.998 | L-shaped |  | | AJL | 1 | 1 | L-shaped | |  |
| DL | 0.991 | 0.997 | L-shaped |  | | QR |  |  |  | |  |
| MJS | 0.993 | 0.995 | L-shaped |  | | RMX | 0.99 | 0.992 | L-shaped | |  |
| NWT | 0.897 | 0.913 | L-shaped |  | | LD | 0.974 | 0.979 | L-shaped | |  |
| SL | 0.949 | 0.966 | L-shaped |  | | RLJ | 0.966 | 0.99 | L-shaped | |  |
| AK | 0.998 | 0.998 | L-shaped |  | | WN | 0.986 | 0.99 | L-shaped | |  |
| LY | 0.988 | 0.988 | L-shaped |  | | RXS | 0.993 | 0.993 | L-shaped | |  |
| SHS | 0.688 | 0.754 | Shifted mode ^b^ | | RMAK | | 1 | 1 | L-shaped | |  |
| LS | 0.852 | 0.852 | Shifted mode | | RML | | 0.997 | 0.997 | L-shaped | |  |

Notes: QR = *Quercus rehderiana*, QS = *Q. spinosa*, QA = *Q. aquifolioides*.

SMM, stepwise mutation model; TPM, two-phase mutation model. *P*-values are shown for Wilcoxon’s sign-rank test under both the SMM and the TPM, along with the shape of the allelic distribution inferred from the mode-shift test. ^a^ Alleles is expected in the absence of a bottleneck, ^b^ Alleles is expected in a population that has gone through a bottleneck.
