# Supplementary material for: Genetic Structure and Evolutionary History of Three Alpine Sclerophyllous Oaks in East Himalaya-Hengduan Mountains and Adjacent Regions
Source: Front Plant Sci. 2016 Nov 11;7:1688. doi: 10.3389/fpls.2016.01688 (PMC5104984; doi:10.3389/fpls.2016.01688)
Supplement: Table S6 — The nine scenarios for four lineages. [file Table6.DOCX]

**Table S6** The nine scenarios for four lineages

| Scenarios |  |
| --- | --- |
| Scenario 1 | *Quercus. aquifolioides* (QA) and a common ancestor of *Q. rehderiana* (QR) and *Q. spinosa* (west lineage, QS1; east lineage, QS2, also see Fig. S3) diverged at time t3, and then QR and the ancestor of QS1 and QS2 diverged at t2, and QS1 and QS2 diverged at time t1. |
| Scenario 2 | QA and a common ancestor of QR, QS1and QS2 diverged at time t2, and then QR and a common ancestor of QS1 and QS2 diverged at the same time, t1. |
| Scenario 3 | The ancestor of QS1, QS2, QR and QA diverged at t2, and then QS1 and QS2 as well as QR and QA diverged at the same time, t1, respectively. |
| Scenario 4 | The ancestor of QS1, QS2, QR and QA diverged at t3, and QA and QR diverged at t2, and QS1 and QS2 diverged at t1. |
| Scenario 5 | The ancestor of QS1, QS2, QR and QA diverged at t3, and QS1 and QS2 diverged at t2, and QA and QR diverged at t1. |
| Scenario 6 | The ancestor of QS1, QS2, and QA diverged at t3, and QR was generated by admixture of QA and the ancestor of QS1 and QS2 at t2, and then QS1 and QS2 diverged at t1. |
| Scenario 7 | The ancestor of QS1, QR, and QA diverged at t3, and QS1 and QR diverged at t2, and the Q2 was generated by admixture of QA and the ancestor of QS1 at t1. |
| Scenario 8 | QA and a common ancestor of QR, QS1 and QS2 diverged at time t3, and QS1 and QR diverged at t2, and then QS2 was generated by admixture of QR andQS1 at t1. |
| Scenario 9 | QA and QS1 diverged at time t3, and QR was generated by admixture of QA and QS1 at t2, and QS2 was generated by admixture of QA and QS1 at t1. |
