# Supplementary material for: Genetic Structure and Evolutionary History of Three Alpine Sclerophyllous Oaks in East Himalaya-Hengduan Mountains and Adjacent Regions
Source: Front Plant Sci. 2016 Nov 11;7:1688. doi: 10.3389/fpls.2016.01688 (PMC5104984; doi:10.3389/fpls.2016.01688)
Supplement: Table S7 — Prior distributions for model parameters used in model comparisons. [file Table7.DOCX]

**Table S7** Prior distributions for model parameters used in model comparisons

| Parameters ^a^ | Prior ^b^ | Parameters ^c^ | Prior ^b^ |
| --- | --- | --- | --- |
| NQS1 | (1.00E+2, 1.00E+6) | N1 | (1.00E+2, 1.00E+6) |
| NQS2 | (1.00E+2, 1.00E+6) | N1b | (1.00E+2, 1.00E+6) |
| NQA | (1.00E+2, 1.00E+6) | N2 | (1.00E+2, 1.00E+6) |
| NQR | (1.00E+2, 1.00E+6) | NA | (1.00E+2, 1.00E+6) |
| NA | (1.00E+2, 1.00E+6) | t1 | (1.00E+2, 1.00E+4) |
| t1 | (1.00E+2, 1.00E+4) | db ^d^ | (1.00E+3, 5.00E+4) |
| t2 | (1.00E+3, 5.00E+4) | t2 | (1.00E+3, 1.00E+5) |
| t3 | (1.00E+3, 1.00E+5) |  |  |
| ra | (0, 1) |  |  |
| Mutation model ^e^ |  |  |  |
| Mean mutation rate | (1.00E-6, 1.00E-3) |  |  |
| Individual locus mutation rate | (1.00E-7, 1.00E-2) |  |  |
| Mean coefficient *P* | (0.1, 0.9) |  |  |
| Individual locus coefficient *P* | (0.01, 0.9) |  |  |
| Mean SNI rate | 0 |  |  |
| Individual locus SNI rate | 0 |  |  |

^a^ QS, QR and QA indicate *Quercus. spinosa*, *Q. rehderiana* and *Q. aquifolioides*, respectively. *P* represents the proportion of multiple step mutations in the generalized stepwise model, GSM. ^b^All priors were uniformly distributed. ^c^ All parameters were used to infer changes of effective population sizes of the three oak species. ^d^ duration of bottleneck. ^e^ The mutation model was used in both ABC analysis.

The unit of timing is generation.
