# Supplementary material for: Genetic Structure and Evolutionary History of Three Alpine Sclerophyllous Oaks in East Himalaya-Hengduan Mountains and Adjacent Regions
Source: Front Plant Sci. 2016 Nov 11;7:1688. doi: 10.3389/fpls.2016.01688 (PMC5104984; doi:10.3389/fpls.2016.01688)
Supplement: Table S8 — The 19 bioclimatic variables used in the full set of variables, with √ indicating the variables used in the customized sets for Q. spinosa (QS), Q. rehderiana (QR), and Q. aquifolioides (QA); the variables used in the reduced set. [file Table8.DOCX]

**Table S8** The 19 bioclimatic variables used in the full set of variables, with √ indicating the variables used in the customised sets for *Q. spinosa* (QS), *Q. rehderiana* (QR) and *Q. aquifolioides* (QA); the variables used in the reduced set.

| Code | Bioclimatic variable | Customised set | | | Reduced set |
| --- | --- | --- | --- | --- | --- |
|  |  | QS | QR | QA |  |
| BIO1 | Annual Mean Temperature | √ |  |  |  |
| BIO2 | Mean Diurnal Range [Mean of monthly (max temp–min temp)] |  |  |  |  |
| BIO3 | Isothermality (BIO2/BIO7) (* 100) | √ | √ |  |  |
| BIO4 | Temperature Seasonality (standard deviation * 100) | √ | √ | √ | √ |
| BIO5 | Max Temperature of Warmest Month | √ | √ |  |  |
| BIO6 | Min Temperature of Coldest Month | √ |  |  |  |
| BIO7 | Temperature Annual Range (BIO5-BIO6) | √ | √ | √ | √ |
| BIO8 | Mean Temperature of Wettest Quarter | √ |  |  |  |
| BIO9 | Mean Temperature of Driest Quarter | √ | √ | √ | √ |
| BIO10 | Mean Temperature of Warmest Quarter | √ |  |  |  |
| BIO11 | Mean Temperature of Coldest Quarter | √ | √ | √ | √ |
| BIO12 | Annual Precipitation | √ |  |  |  |
| BIO13 | Precipitation of Wettest Month | √ |  |  |  |
| BIO14 | Precipitation of Driest Month | √ |  |  |  |
| BIO15 | Precipitation Seasonality (Coefficient of Variation) | √ | √ | √ | √ |
| BIO16 | Precipitation of Wettest Quarter |  |  |  |  |
| BIO17 | Precipitation of Driest Quarter | √ | √ |  |  |
| BIO18 | Precipitation of Warmest Quarter | √ | √ | √ | √ |
| BIO19 | Precipitation of Coldest Quarter | √ | √ | √ | √ |
