# Supplementary material for: Genetic Structure and Evolutionary History of Three Alpine Sclerophyllous Oaks in East Himalaya-Hengduan Mountains and Adjacent Regions
Source: Front Plant Sci. 2016 Nov 11;7:1688. doi: 10.3389/fpls.2016.01688 (PMC5104984; doi:10.3389/fpls.2016.01688)
Supplement: Table S9 — Posterior median estimate and 95% highest posterior density interval (HPDI) for demographic parameters of an contraction-expansion model based on the nuclear multilocus microsatellite data of three oak species, respectively. [file Table9.DOCX]

**Table S9** Posterior median estimate and 95% highest posterior density interval (HPDI) for demographic parameters of an contraction-expansion

model based on the nuclear multilocus microsatellite data of three oak species, respectively.

|  | Parameters | N1 ^a^ | N1b ^b^ | NA ^c^ | t1 (generations) | t2 (generations) | *μ* | *P* |
| --- | --- | --- | --- | --- | --- | --- | --- | --- |
| QS | Median | 5.72E+05 | 7.41E+02 | 5.31E+05 | 3.03E+03 | 3.17E+04 | 4.49E-04 | 0.269 |
|  | Lower_bound | 8.90E+04 | 1.00E+02 | 9.55E+04 | 3.39E+02 | 9.18E+03 | 6.24E-05 | 0.157 |
|  | Upper_bound | 9.60E+05 | 7.09E+03 | 9.51E+05 | 8.98E+03 | 4.82E+04 | 9.88E-04 | 0.300 |
| QA | Median | 5.43E+05 | 2.00E+03 | 4.84E+05 | 3.67E+03 | 3.22E+04 | 2.46E-04 | 0.259 |
|  | Lower_bound | 7.74E+04 | 2.19E+02 | 8.78E+04 | 3.99E+02 | 9.85E+03 | 2.37E-05 | 0.145 |
|  | Upper_bound | 9.58E+05 | 2.56E+04 | 9.42E+05 | 9.20E+03 | 4.83E+04 | 9.20E-04 | 0.300 |
| QR | Median | 5.72E+05 | 1.56E+03 | 4.60E+05 | 3.53E+03 | 3.21E+04 | 3.05E-04 | 0.254 |
|  | Lower_bound | 8.94E+04 | 1.89E+02 | 7.71E+04 | 4.15E+02 | 9.72E+03 | 3.37E-05 | 0.140 |
|  | Upper_bound | 9.63E+05 | 1.96E+04 | 9.36E+05 | 9.17E+03 | 4.83E+04 | 9.46E-04 | 0.300 |

^a , c^ The current and ancient population sizes of *Quercus. spinosa*, *Q. aquifolioides* and *Q. rehderiana*, respectively. ^b^ populations sizes between NA and N1.
