# Supplementary material for: Genetic Structure and Evolutionary History of Three Alpine Sclerophyllous Oaks in East Himalaya-Hengduan Mountains and Adjacent Regions
Source: Front Plant Sci. 2016 Nov 11;7:1688. doi: 10.3389/fpls.2016.01688 (PMC5104984; doi:10.3389/fpls.2016.01688)
Supplement: Note S1 — Description of the oak species and their habitats in our study. [file Presentation1.PDF]

Note S1 Description of the oak species and their habitats in our study

In the present study, we focused on three species of *Quercus* Group Ilex (synonyms *Quercus* subgenus *Heterobalanus*) (Menitsky, 2005), i.e. *Quercus. spinosa* (syn. *Q. bullata*, *Q. gilliana*, *Q. tatakaensis*, *Q. taiyunensis*, *Q. ilex* Linnaeus var. *spinosa*, *Q. semecarpifolia* Smith var. *spinosa*,), *Q. aquifolioides* and *Q. rehderiana* (syn. *Q. longispica*, *Q. pseudosemecarpifolia*, *Q. semecarpifolia* Smith var. *glabra*, *Q. semecarpifolia* var. *longispica*). *Q. spinosa* is shade-intolerant, and occurs as an understorey shrub/tree in warm- or cool-temperate *Fagus* forests with low- to mid-elevation in China (*ca*. 900-3800 m above sea level (*a.s.l*)), commonly growing on the sunny slopes and cliffs. *Q. aquifolioides* is shade-intolerant, while *Q. rehderiana* is moderately shade-tolerant; both species are endemic to China's EH-HM region, and are usually trees with the height of 6-15 m, occurring in cold-dry or subhumid environments at high altitudes (*ca*. 2,000 to 4,500 *a.s.l* and 1,500 to 4,000 *a.s.l*, respectively) in Southwest China (Wu *et al.*, 1999).

The three closely related species (i.e. *Quercus spinosa*, *Q. aquifolioides* and *Q. rehderiana*) have distinguish morphological characterizations with flowers, color and degree of density of stellate hairs on leaf, shape of cupule, bracteole and acorn (Wu *et al.*, 1999). Although their distribution ranges are overlapping, in our filed survey, their microhabitats are different (*e.g.* occur in different slopes in the same mountain, shade-tolerant or shade-intolerant, in other words, they are allopatric or parapatric).

References

Menitsky Û.L. (2005) *Oaks of Asia*. Enfield: Science Publisher,.

Wu, Z.Y., Raven, P. & Hong, D.Y. (1999) *Flora of China. Cycadaceae through Fagaceae*, *vol. 4*. Beijing and St. Louis: Science Press, and Missouri Botanical Garden Press.

Note S2 methodological details for sequences and microsatellite genotyping

DNA extraction, PCR and sequence modification

Genomic DNA was extracted using a modified CTAB protocol (Murray & Thompson, 1980). We selected *ITS*4 and *ITS*5, *psb*A-*trn*H, *psb*B-*psb*F and *matK* for amplifying the entire internal transcribed spacer (ITS) and three intergenic spacer (IGS) regions of cpDNA (White *et al*., 1990; Hamilton, 1999; Piredda *et al.*, 2011), the sequences and annealing temperature of primers please see below in Table 1. PCR for chloroplast and ITS required an initial denaturation step of 3 min at 94 ℃, followed by 40 cycles of 30 sec at 94 ℃ denaturation, 45 sec at 52 - 58 ℃ (52 ℃ for ITS, 54 ℃ for *psb*A-*trn*H and *mat*K, 58 ℃ for *psb*B-*psb*F) annealing and 90 sec at 72 ℃ extension, and a final extension at 72 ℃ for 10 min. The PCR products were purified and sequenced by Sangon Biotechnology (Shanghai, China). DNA sequences were aligned with BIOEDIT v7.0.9 (Hall, 1999) and CLUSTAL W as implemented in MEGA v5.0 (Tamura *et al.*, 2011) with further manual modifications. All sequences generated in this study have been deposited into GenBank under the accession numbers KT997345–KT997421.

Nuclear microsatellite genotyping

All samples except for one sample from Taiwan (609 individuals in total, see Table S1 in Appendix S1) were genotyped at the following genomic microsatellite loci: 1P10, 2P24, 3A05, 3D15, MSQ3, MSQ13, QrZAG11, QrZAG20, QrZAG30, QpZAG9, QpZAG15, QpZAGA46 (Dow *et al.*, 1995; Steinkellner *et al.*, 1997; Kampfer *et al.*, 1998; Durand *et al.*, 2010), the sequences and annealing temperature of primers please see below in Table 2. Microsatellite loci were amplified with the following cycle: 5 min initial denaturation at 95 ℃; 30 cycles of 30 sec at 95 ℃, 30 sec at the optimized annealing temperature, 45 sec of elongation at 72 ℃, and a final extension at 72 ℃ for 10 min. The initial PCR products were separated on 8% non-denaturing polyacrylamide gel (280 V, 50 W), and visualized by 0.1% silver nitrate staining. Allele size was determined by comparing with the DNA size ladder. Then we selected individuals per population which had different allele sizes using automatic capillary electrophoresis system and SSR genotyping scoring were subsequently analysed using GENEMAPPER software version 3.7 (Applied Biosystems) and checked visually twice. According to the latter results determined by automatic capillary electrophoresis system, we adjusted our primary results. The nSSR data of these three oak species are provided in Table S10.

References

Dow, B.D., Ashley, M.V., and Howe, H.F. (1995) Characterization of highly variable (GA/CT) n microsatellites in the bur oak, *Quercus macrocarpa*. *Theor. Appl. Genet.* 91, 137-141. doi: 10.1007/BF00220870.

Durand, J., Bodenes, C., Chancerel, E., Frigerio, J-M., Vendramin, G., Sebastiani, F., Buonamici, A., Gailing, O., Koelewijn, H-P., Villani, F., et al. (2010) A fast and cost-effective approach to develop and map EST-SSR markers: oak as a case study. *BMC Genomics*, 11, 570. doi: 10.1186/1471-2164-11-570.

Hall, T.A. (1999) BioEdit: a user-friendly biological sequence alignment editor and analysis program for Windows 95/98/NT. *Nucleic Acids Symp. Ser.* 41, 95-98.

Hamilton, M. (1999) Four primer pairs for the amplification of chloroplast intergenic regions with intraspecific variation. *Mol. Ecol.* 8, 521-523.

Kampfer, S., Lexer, C., Glössl, J., and Steinkellner, H. (1998) Characterization of (GA)n microsatellite loci from *Quercus robur*. *Hereditas*, **129**, 183-186. doi: 10.1111/j.1601-5223.1998.00183.x

Piredda, R., Simeone, M.C., Attimonelli, M., Bellarosa, R., and Schirone, B. (2011) Prospects of barcoding the Italian wild dendroflora: oaks reveal severe limitations to tracking species identity. *Mol. Ecol. Resour.* 11, 72-83. doi: 10.1111/j.1755-0998.2010.02900.x

Steinkellner, H., Fluch, S., Turetschek, E., Lexer, C., Streiff, R., Kremer, A., Burg, K., and Glössl, J. (1997) Identification and characterization of (GA/CT)n - microsatellite loci from *Quercus petraea*. *Plant Mol. Biol.*, 33, 1093-1096.

Tamura, K., Peterson, D., Peterson, N., Stecher, G., Nei, M., and Kumar, S. (2011) MEGA5: Molecular evolutionary genetics analysis using maximum likelihood, evolutionary distance, and maximum parsimony methods. *Mol. Biol. Evol.* 28, 2731-2739. doi: 10.1093/molbev/msr121

White, T.J., Bruns, T., Lee, S., and Taylor, J. (1990) “Amplification and direct sequencing of fungal RNA genes for phylogenetics.” in *PCR Protocols: A Guide to Methods and Applications* ed. M.A. Innis, D.H. Gelfand, J.J. Sninsky, and T.J. White. (New York: Academic Press), 315-322.

Table 1 The primer sequences and annealing temperatures for cpDNA and nrDNA used in this study

Primer	Forward primer	Backward primer	Annealing temperature (℃)
*ITS*4-*ITS*5	TCCTTCCGCTTATTGATATGC	GGAAGGAGAAGTCGTAACAAGG	52
*psb*A-*trn*H	CGAAGCTCCATCTACAAATGG	ACTGCCTTGATCACATTGGC	54
*mat*K	ACCCAGTCCATCTGGAAATCTTGGTTC	CGTACAGTACTTTTGTGTTTACGAG	54
*psb*B-*psb*F	GTTTACTTTTGGGCATGCTTCG	CGCAGTTCGTCTTGGACCAG	58

Table 2 The nSSR primer sequences and annealing temperatures used in this study

Primer	Sequence (5'-3')	Annealing temperature (℃)	Source/Source species
3A05	F-AACGTGACCTCTCTCACAGC, R-AGTGCTGGAGTGCTCATGG	64	Durand et al., 2010 (*Q. robur*)
3D15	F-GGTGGTGGCAGATACACTGG, R-GACTCAGACAACCAACTTCAGG	64	Durand et al., 2010 (*Q. robur*)
1P10	F-ATTTCTGATGCAGGGTGTCG, R-TAGGCCAAGGACCAGAGACC	66	Durand et al., 2010 (*Q. robur*)
2P24	F-GCAAGAGATCACACACAAACTAGC, R-CTTTGGGTTCACCAAACAGC	66	Durand et al., 2010 (*Q. robur*)
ZAG30	F-TGCTCCGTCATAATCTTGCTCTGA, R-GCAATCCTATCATGCACATGCACAT	50	Kampfer et al., 1998 (*Q. robur*)
ZAG11	F-CCTTGAACTCGAAGGTGTCCTT, R-GTAGGTCAAAACCATTGGTTGACT	55	Kampfer et al., 1998 (*Q. robur*)
ZAG15	F-CGATTTGATAATGACACTATGG, R- CATCGACTCATTGTTAAGCAC	48	Steinkellner et al.,1997 (*Q. petraea*)
ZAG9	F- GCAATTACAGGCTAGGCTGG, R- GTCTGGACCTAGCCCTCATG	55	Steinkellner et al.,1997 (*Q. petraea*)
ZAG20	F-CCATTAAAAGAAGCAGTATTTTGT, R-GCAACACTCAGCCTATATCTAGAA	53	Kampfer et al., 1998 (*Q. robur*)
ZAG46	F- CCCCTATTGAAGTCCTAGCCG, R- TCTCCCATGTAAGTAGCTCTG	53	Steinkellner et al., 1997 (*Q. petraea*)
MSQ13	F-ACACTCAGACCCACCATTTTTCC, R- TGGCTGCACCTATGGCTCTTAG	65	Dow et al., 1995 (*Q. macrocarpa*)
MSQ4	F-TCTCCTCTCCCCATAAACAGG, R- GTTCCTCTATCCAATCAGTAGTGAG	50	Dow et al., 1995 (*Q. macrocarpa*)

F-: Forward-; R-: Reverse-
